# Sustained *in vivo* signaling by long-lived IL-2 induces prolonged increases of regulatory T cells

**DOI:** 10.1016/j.jaut.2014.10.002

**Published:** 2015-01

**Authors:** Charles J.M. Bell, Yongliang Sun, Urszula M. Nowak, Jan Clark, Sarah Howlett, Marcin L. Pekalski, Xin Yang, Oliver Ast, Inja Waldhauer, Anne Freimoser-Grundschober, Ekkehard Moessner, Pablo Umana, Christian Klein, Ralf J. Hosse, Linda S. Wicker, Laurence B. Peterson

**Affiliations:** a JDRF/Wellcome Trust Diabetes and Inflammation Laboratory, Department of Medical Genetics, Cambridge Institute for Medical Research, NIHR Cambridge Biomedical Research Centre, University of Cambridge, Cambridge CB2 OXY, United Kingdom; b Former Roche Site of Pharmaceutical Research and Early Development, Discovery Inflammation, Nutley, NJ 07110, USA; c Roche Pharmaceutical Research and Early Development, Oncology Discovery & Translational Area, Roche Innovation Center Zurich, Wagistrasse 18, CH-8952 Schlieren, Switzerland; d Roche Pharmaceutical Research and Early Development, Large Molecule Research, Roche Innovation Center Zurich, Wagistrasse 18, CH-8952 Schlieren, Switzerland

**Keywords:** Cytokine therapy, Autoimmunity, Graft versus host disease, IL-2 fusion proteins, Regulatory T cells, GVHD, graft versus host disease, PK, pharmacokinetics

## Abstract

Regulatory T cells (Tregs) expressing FOXP3 are essential for the maintenance of self-tolerance and are deficient in many common autoimmune diseases. Immune tolerance is maintained in part by IL-2 and deficiencies in the IL-2 pathway cause reduced Treg function and an increased risk of autoimmunity. Recent studies expanding Tregs *in vivo* with low-dose IL-2 achieved major clinical successes highlighting the potential to optimize this pleiotropic cytokine for inflammatory and autoimmune disease indications. Here we compare the clinically approved IL-2 molecule, Proleukin, with two engineered IL-2 molecules with long half-lives owing to their fusion in monovalent and bivalent stoichiometry to a non-FcRγ binding human IgG1. Using nonhuman primates, we demonstrate that single ultra-low doses of IL-2 fusion proteins induce a prolonged state of *in vivo* activation that increases Tregs for an extended period of time similar to multiple-dose Proleukin. One of the common pleiotropic effects of high dose IL-2 treatment, eosinophilia, is eliminated at doses of the IL-2 fusion proteins that greatly expand Tregs. The long half-lives of the IL-2 fusion proteins facilitated a detailed characterization of an IL-2 dose response driving Treg expansion that correlates with increasingly sustained, suprathreshold pSTAT5a induction and subsequent sustained increases in the expression of CD25, FOXP3 and Ki-67 with retention of Treg-specific epigenetic signatures at *FOXP3* and *CTLA4*.

## Highlights

• Fusion of IL-2 to human IgG1 extends IL-2 half-life and efficiency of Treg induction.
• Long-lived IL-2 efficacy allows for lower doses thereby increasing Treg specificity.
• Sustained pSTAT5a induction in Tregs by long-lived IL-2 correlates with Treg expansion.
• Increased stoichiometry, IgG-(IL-2)_2_, increases affinity and *in vivo* effects on Tregs

## 1 Introduction

CD4^+^CD25^+^CD127^lo^ Tregs are crucial for the maintenance of self-tolerance, they constitutively express FOXP3, a transcription factor critical for Treg differentiation and function and have suppressive activity associated with expression of CTLA-4, IL-10 and TGFβ [1–4]. The FOXP3 gene is mutated in *scurfy* mice, which develop a lethal autoimmune syndrome, and in patients with immune dysregulation polyendocrinopathy, enteropathy, X-chromosome linked syndrome (IPEX) resulting in the breakdown of self-tolerance and the development of autoimmune disease [5].

Genetic susceptibility to several common autoimmune diseases maps to the IL-2RA gene region as well as other genes involved in IL-2 signaling and Treg function [6]. IL-2 plays a major role in the activation and function of Tregs and effector T cells (Teff): IL-2 binds the IL-2 receptor inducing phosphorylation of Janus-activated kinases leading to the activation of multiple downstream signaling pathways and altered gene expression. The signal transducer and activator of transcript (STAT) family of transcription factors are part of one pathway activated by the Janus-activated kinases and are critical for the upregulation of FOXP3 and CD25 (the α chain of the IL-2 receptor) in Tregs [7,8]. A deficiency in IL-2 production or lack of IL-2 responsiveness preferentially leads to a loss of Treg function and an increase in autoimmunity [9–12]. Tregs constitutively express the trimeric high affinity receptor for IL-2 (IL-2Rαβγ) at higher levels than CD4^+^ and CD8^+^ Teff cells, NK cells and eosinophils [13–16]. IL-2 is essential for Treg survival [17–19] and induction of STAT5a signaling occurs at lower doses of IL-2 in Tregs than in Teff [16]; hence ultra-low dose IL-2 could stimulate preferential activation and promote the survival of Tregs *in vivo*. Proleukin is a recombinant IL-2 traditionally used at high doses for cancer therapy that has recently been used with remarkable success at lower doses in graft-versus-host disease (GVHD) and hepatitis C-induced vasculitis with therapeutic responses correlating with increased Treg frequencies [20,21]. These studies led to ultra-low dose Proleukin as prophylaxis in young GVHD patients [22], mechanistic studies and trials testing Proleukin in healthy volunteers [23] and patients with autoimmune disease (NCT01840046, NCT02084238, NCT01827735, NCT01988506, NCT01862120 [24,25]).

Proleukin has a short pharmacokinetic (PK) half-life [26] making it difficult to maintain low Treg-specific blood levels since its frequent, often daily, dosing produces peak blood levels with the potential to activate non-Tregs such as CD4^+^ Teff cells, NK cells and eosinophils. Thus methods to increase the half-life of IL-2, thereby enabling lower doses of IL-2 to be delivered, are therapeutically desirable and molecular engineering approaches to increase cytokine half-lives and bioavailability have been recently reviewed, *e.g.* a variety of fusion proteins including IgG, antibody complexes and PEGylation [27]. Several clinical trials for cancer are currently using IL-2 covalently attached to antibodies that are targeted to molecules enhancing their tumor-specific distribution, *e.g.* tenascin C, fibronectin and DNA/histones [27]. In contrast, here we utilize a non-targeted IgG molecule as a half-life enhancer to produce IL-2 fusion proteins that can potentially be used in a variety of autoimmune and inflammatory diseases. The use of flexible spacers to link IL-2 to the Fc region of IgG and the ability to generate IgG molecules having one or two IL-2 molecules per IgG allowed us to test the possibility that the avidity of the IL-2 fusion protein could be increased by providing suitable conformation and spacing of two IL-2 molecules for engaging two high affinity IL-2 receptors simultaneously on Tregs. This strategy has the potential to provide even greater Treg specificity by further lowering the dose of IL-2 thereby reducing *C*_max_-associated effects and minimizing occurrences of blood levels below the threshold for Treg activation. Ultimately such molecules would be expected to have sustained effects on Tregs and result in improved therapeutic benefits. Here we test these hypotheses using cynomolgus as a preclinical translational species. The IL-2 fusion proteins were given at single ultra-low doses and preferentially activated and increased Tregs up to two weeks post treatment. Similar responses could be generated with multiple doses, but not with single doses, of Proleukin. These attributes enable ultra-low doses of IL-2 with less frequent administration to maintain long-term enhancement of Tregs.

## 2 Materials and methods

### 2.1 Cynomolgus monkeys and NOD.*scid Il2ra*^*null*^ mice

Healthy adult male and female cynomolgus monkeys were used in all tests; they were used in only one test and had never been given a human protein. With the exception of epigenetic studies requiring males, all cynomolgus were randomly assigned to treatment groups without regard to age or gender. All monkeys were in a colony at Hoffmann-La Roche (Nutley, NJ), were between 4 and 11 years of age and weighed between 4 and 13 kg. All procedures were performed with adherence to the NIH Guide for the Care and Use of Laboratory Animals and were approved by the Roche Institutional Animal Care and Use Committee and the Roche Ethics Committee for Animal Welfare. Subcutaneous (SQ) injections of 5 ml (1 ml in each of five sites) were performed under anesthesia in the lateral dorsum. Vehicle treatments were 0.5% sterile cynomolgus serum in PBS. The safety, efficacy and biologic responses to the IL-2 fusion proteins were monitored in blood samples taken during the studies using routine clinical hematology and chemistry analyses with an Advia Automated Hematology Analyser (Siemens). Lymphocyte and eosinophil numbers were obtained from the clinical hematology complete blood count and in conjunction with flow cytometry data were used to calculate absolute numbers (per mm^3^ or per ml) of specific cell subsets.

NOD.*scid* and NOD.*scid Il2ra*^*null*^ mice were obtained from Taconic Farms (Germantown, NY) and housed under specific pathogen-free conditions at Hoffmann-La Roche (Nutley, NJ). The Roche Institutional Animal Care and Use Committee approved all experimental procedures. The generation of NOD.*scid Il2ra*^*null*^ mice was performed under contract at Taconic Farms; briefly, heterozygous B6.129S4-*Il2ra*^*tm1Dw*^/J mice were backcrossed to NOD/MrkTac mice nine times and then backcrossed twice to NOD.*scid* mice. Mice heterozygous for the *Il2ra*^*null*^ knockout and homozygous for *scid* were intercrossed to generate homozygous NOD.*scid Il2ra*^*null*^ mice. The mice contain at maximum a 3.131 Mb 129 congenic interval containing the *IL2ra* knockout, which is defined by the markers rs27117457 (proximal out), rs27100797 (proximal in), Chr2 12.60 (distal in) and rs13476352 (distal out). A 1449 SNP marker panel across 19 autosomes and the X chromosome, averaging a genetic interval of 5 Mbps (1449 marker panel, Taconic) was used to verify NOD homozygosity throughout the remainder of the genome.

### 2.2 IL-2

Proleukin^®^ (human recombinant IL-2, Prometheus Laboratories, San Diego CA) was purchased from a local pharmacy and diluted with 1.2 ml water for injection with each 1 ml solution containing 1.1 mg (18 × 10^6^ IU), a new vial of Proleukin was used for each day of treatment. The IL-2 fusion proteins, IgG-IL-2 and IgG-(IL-2)_2_, were prepared at Hoffmann-La Roche (patent WO2014/023752A1). In brief, the molecules consist of a human IgG1 with V-domain germline sequences and an engineered short VH CDR3 that has no known antigen-binding properties on human cells or tissues. Specific point mutations in the Fc-portion of the IgG1 (P329G L234A L235A) rendered it effector silent by abolishing FcRγ binding while leaving normal FcRn function intact. Each IgG1 was engineered to have one or two wild-type human IL-2 molecules covalently fused at their N-terminal amino acid to the C-terminus of one or both of the IgG1 heavy chains (omitting the C-terminal lysine) via a G4S-peptide linker, *i.e.* IgG-IL-2 and IgG-(IL-2)_2_. Knobs-into-holes technology was used to engineer the monovalent IgG-IL-2 [28,29]. The molecular weights used to calculate pM and pmol/kg are the following: Proleukin (15,300), IgG-IL-2 (159,088) and IgG-(IL-2)_2_ (175,350).

### 2.3 Surface plasmon resonance

Binding affinities were measured by Biacore surface plasmon resonance on human, cynomolgus and murine IL-2Rα and IL-2Rβγ on a Biacore T200 (GE Healthcare). Monomeric his-tagged IL2Rα was chemically immobilized by amine coupling on CM5 chips. A 2-fold dilution series (0.41–300 nM) of IgG-IL-2 and IgG-(IL-2)_2_ was injected over the chip surface for 90 s and the dissociation was monitored for 3 min. The surface was regenerated after each injection by washing with 10 mM glycine pH 1.5 for 60 s. Due to the fast association and dissociation rates, the binding curves were fitted using a steady state model (BIAevaluation software). To measure binding to IL2Rβγ, a heterodimeric Fc fusion was generated by applying knobs-into-holes technology [28,29]. The IL2Rβ and γ chains were fused to the hole or knob chain of the Fc, respectively, and co-expressed to obtain the preformed recombinant heterodimer also carrying an avi-tag for site-specific biotinylation. These biotinylated IL2Rβγ heterodimers were immobilized on streptavidin chips. A 2-fold dilution series of the cytokine fusion constructs (1.2–300 nM) was injected over the chip surface for 2 min and the dissociation was monitored for 10 min (for the two highest concentrations) to observe a measurable decay of these high-affinity complexes. The surface was regenerated after each injection by washing with 3 M MgCl_2_. The binding curves were fitted globally using a 1:1 interaction model (BIAevaluation software), despite the bivalent binding of the IgG-(IL-2)_2_ which is considered an ‘apparent’ *K*_D_. The very slow off-rates result in a low *K*_D_ in the pM range that approaches the limit of the instrument.

### 2.4 *In vitro* human and cynomolgus blood pSTAT5a

Human blood from healthy adults was collected with informed consent from the Cambridge BioResource with ethical approval by the Peterborough and Fenland Local Research Ethics Committee (05/Q0106/20).

The effects of Proleukin, IgG-IL-2, and IgG-(IL-2)_2_ on the induction of pSTAT5a were assessed in human CD4^+^ Treg subsets, naïve and memory conventional CD4^+^ T cells, memory conventional CD8^+^ T cells, CD45RA^+^ CD8^+^ T cells, NKT cells and NK cells. IgG-IL-2 and IgG-(IL-2)_2_ were assessed in similar cynomolgus cell subsets. All subsets were characterized in a single tube for each dose of the different IL-2 molecules. Briefly, blood from a healthy human adult donor or cynomolgus monkey was collected into heparinized tubes. Various concentrations of Proleukin or IL-2 fusion proteins were added to 0.5 ml of blood and incubated at 37 °C. After 30 min the blood was lysed and fixed using pre-warmed lyse/fix buffer (Becton Dickinson Biosciences) for 10 min at 37 °C, washed 2× with PBS containing 0.2% BSA followed by permeabilization with −20 °C pre-cooled methanol (Sigma, Biotech grade) for 20 min on ice. The cells were then extensively washed 4× with PBS containing 0.2% BSA before FACS staining was performed using a panel of fluorescent antibodies to distinguish different lymphocyte and NK cell subpopulations and pSTAT5a status. The antibodies used for staining human blood cells were CD4-Alexa Fluor 700 (clone RPA-T4), CD3-PerCP/Cy5.5 (UCHT1), CD45RA-PE/Cy7 (HI100), CD8-Brilliant Violet 605 (RPA-T8), CD56-Brilliant Violet 421 (HCD56), FOXP3-PE (259D) (all from BioLegend), CD25-APC (clones M-A251 & 2A3) and pSTAT5a-Alexa Fluor 488 (pY694) (Becton Dickinson Biosciences). The antibodies used for staining cynomolgus blood cells were FOXP3-Alexa Fluor^®^ 647 (clone 259D, BioLegend), CD4-V500 (clone L200, BD Biosciences), CD45RA-V450 (clone 5H9, BD Biosciences), CD25-PE (clone 4E3, eBioscience), pSTAT5a-Alexa Fluor^®^ 488 (clone 47, BD Biosciences), and CD3-PerCP-Cy5.5 (clone SP34-2, BD Biosciences). Samples were acquired using an LSRFortessa cell analyzer (Becton Dickinson) and data analyzed using FlowJo software (TreeStar). After gating on lymphocytes and excluding doublets, human Tregs were defined as CD3^+^CD4^+^FOXP3^+^ and subdivided as CD3^+^CD4^+^CD45RA^−^FOXP3^+^ (memory Treg) and CD3^+^CD4^+^CD45RA^+^FOXP3^+^ (naïve Treg). Conventional CD4^+^ T cells were defined as CD3^+^CD4^+^CD45RA^+^ (naïve) and CD3^+^CD4^+^CD45RA^−^ (memory). CD8^+^ T cells were defined as CD3^+^CD8^+^CD45RA^−^ (memory) and CD3^+^CD8^+^CD45RA^+^ (mainly composed of naïve CD8^+^ Teff but also containing terminally differentiated CD8^+^ memory Teff). Human NKT cells were defined as CD3^+^CD56^+^ and NK cells were defined as CD3^−^CD56^+^ or CD3^−^CD56^bright^. Similar to humans, flow cytometry readily distinguished cynomolgus CD4^+^FOXP3^+^ Tregs into naïve (FOXP3^+^CD45RA^+^) and memory (FOXP3^+^CD45RA^−^) subsets with each subset of CD4^+^CD25^+^FOXP3^+^ Tregs expressing high levels of the high affinity IL-2 receptor CD25. Cynomolgus conventional CD4^+^ memory T effector cells can similarly be identified in blood as CD4^+^FOXP3^−^CD45RA^−^ cells with approximately 80% of cells being CD25^−^ and 20% being CD25^+^. When memory Teff cells were subdivided into CD25^−^ and CD25^+^ subsets, the response to IL-2 was more sensitive in the CD25^+^ subset than the CD25^−^ subset. Cynomolgus NK cells were identified as CD3^−^CD8^+^CD45RA^hi^. Intracellular pSTAT5a levels were quantified in all cell subsets at all doses.

### 2.5 *Ex vivo* cynomolgus flow cytometry and pSTAT5a

Similar to human blood, cell surface and intracellular markers were used to identify regulatory T cell subsets and conventional T cells in whole blood from cynomolgus monkeys. Blood samples were collected in heparin before and at various times after treatment with Proleukin or the IL-2 fusion proteins. Blood samples were lysed and fixed with pre-warmed BD Lyse/Fix buffer (BD Biosciences). After washing, cells to be stained with the intracellular panel were permeabilized with 1 ml methanol for 30 min on ice. Samples were washed three times and stained with a panel of FOXP3-Alexa Fluor^®^ 647 (clone 259D, BioLegend), CD4-V500 (clone L200, BD Biosciences), CD45RA-V450 (clone 5H9, BD Biosciences), CD25-PE (clone 4E3, eBioscience), pSTAT5a-Alexa Fluor^®^ 488 (clone 47, BD Biosciences) and Ki-67-PerCP-Cy5.5 (clone B56, BD Biosciences) for 1 h at 4 °C. Blood samples were also stained with a surface panel, which consisted of CD3-Alexa Fluor^®^ 488 (clone SP34-2, BD Biosciences), CD16-APC (clone 3G8, BD Biosciences), CD8-V500 (clone SK1, BD Biosciences) and CD122-PE (clone Mik beta3, BD Biosciences).

### 2.6 IL-2 and sCD25 immunoassays

The PK properties of IL-2 fusion proteins were evaluated in adult cynomolgus monkeys injected IV with a short bolus of sterile IgG-IL-2 or IgG-(IL-2)_2_ in PBS containing 0.5% cynomolgus serum and bled at various times after injection, ranging from 30 min to 72 h. Human IL-2 was assessed in the cynomolgus serum samples using mouse anti-human IL-2 mAb (clone 5344.111, BD Pharmingen) to coat 96-well plates in order to immobilize the human IL-2. Human IL-2 was detected using biotinylated mouse anti-human IL-2 mAb (clone B33-2, BD Pharmingen). IL-2 binding was quantified using Eu^++^-conjugated streptavidin. The lower limit of detection using cynomolgus serum was 0.05 ng/ml of IL-2. Normal cynomolgus serum had no detectable IL-2 (<0.05 ng/ml). Monoclonal antibody reagents for human sCD25 known to cross react with cynomolgus sCD25 were used in a sandwich immunoassay using capture (MAB623, R&D Systems) and biotinylated detection (BAF223, R&D Systems) antibodies using Eu^++^-conjugated streptavidin to detect bound sCD25.

### 2.7 DNA demethylation of FOXP3 and CTLA4

The demethylation signatures of *FOXP3* and *CTLA4* were tested in sorted CD4^+^ T cell subsets before and after treatment with IgG-IL-2 (157 pmol/kg, *n* = 4) and IgG-(IL-2)_2_ (34 pmol/kg, *n* = 4). Adult male cynomolgus whose weights ranged from 9.1 to 10.9 kg were used in the study. Treatments were administered SQ 3 weeks after baseline blood collections and post-IL-2 treatment bloods were collected 4–5 days later when Tregs were maximal. PBMCs from 30 ml of blood were sorted using a FACSAria (Becton Dickinson) into relevant CD4^+^ subsets including Tregs (CD4^+^CD25^+^CD127^low^), memory T effectors (CD4^+^CD45RA^−^), naïve T effectors (CD4^+^CD45RA^+^), and CD4^+^CD25^−^CD127^−^ cells. In post-treatment blood we were able to sort additional subsets of Tregs that were CD25^hi^ (CD4^+^CD25^hi^CD127^low^) or CD25^int^. In normal blood CD25^hi^ cells represent less than 1% of the total Tregs were there were insufficient numbers to sort.

Sorted cell subsets were frozen in aliquots of 100,000 cells and stored as dry pellets at −80 °C to allow processing of all samples together. Cell pellets obtained from sorting were thawed and DNA was extracted and bisulfite treated in a single step using Epitect Fast Lyse All kit (Qiagen). 5 ng (in 2 μl) of bisulfite DNA was used in a 20 μl first round duplex PCR containing 10 μl Multiplex PCR kit (Qiagen), 6 μl water, 0.5 μl FOXP3 forward primer tgtaaaacgacggccagtTTTAGAAGTTGTATGGGGGATGTT, 0.5 μl FOXP3 reverse primer caggaaacagctatgaccAAAATATCTACCCTCTTCTCTTCCTC, 0.5 μl CTLA-4 forward primer tgtaaaacgacggccagtGGGTTTGGTTATGAAGGAGTATGA and 0.5 μl CTLA-4 reverse primer caggaaacagctatgaccTTCACTTAATTTCCACTAAAAATACCC. First round PCR cycling was 95 °C for 15 min followed by 20 cycles of 95 °C for 30 s, 60 °C for 90 s and 72 °C for 60 s, followed by 10 min at 72 °C. PCR product was purified using AMPure XP beads (Becton Coulter) according to manufacturer's instructions, and eluted in 20 μl of water. A second round of PCR was performed that added a unique index sequence to the beginning of each sample, through a 15 μl PCR containing 7.5 μl of Multiplex PCR kit (Qiagen), 6.5 μl of first round PCR product and 1 μl of index primer. Second round PCR cycling conditions were 95 °C for 15 min followed by 7 cycles of 95 °C for 30 s, 54 °C for 90 s and 72 °C for 60 s, followed by 5 min at 72 °C. The PCR products were purified using AMPure XP beads and eluted in 15 μl of water, and 4 μl was used to quantify the amount of PCR product using a Shimadzu Multina. Equal molar amounts of each sample were pooled to make the sequencing library which was quantified using a Kapa Illumina library quantification kit. The library was sequenced using an Illumina MiSeq with v3 reagents and 2 × 300 bp paired-end reads. The sequence data were de-multiplexed using a bespoke python script and the Cutadapt program was used to remove sequences generated from sequencing adaptors. Forward and reverse reads were merged using FLASH and the sequence of each methylation site was extracted.

### 2.8 Data analysis

Prism 6 (GraphPad Software, Inc.) was used to calculate area under the curve (AUC); each set of data provided individual baseline *Y*-values and by definition, all peaks were above baseline. Unless noted, all data are shown as the mean ± SEM and *p* values calculated with Prism 6.

## 3 Results

### 3.1 Increased avidity enhances *in vitro* biologic activities

Human and cynomolgus whole blood assays measuring cell-specific increases in pSTAT5a and Biacore surface plasmon resonance assessing binding to IL-2 receptor components were used to characterize the cell activation potencies and binding affinities of IgG-IL-2 and IgG-(IL-2)_2_. For reference, the IL-2 fusion proteins are depicted (Supplemental Fig. 1) and the flow cytometry gating used to identify specific cynomolgus blood cell subsets is shown (Supplemental Fig. 2).

**Fig. 1:**
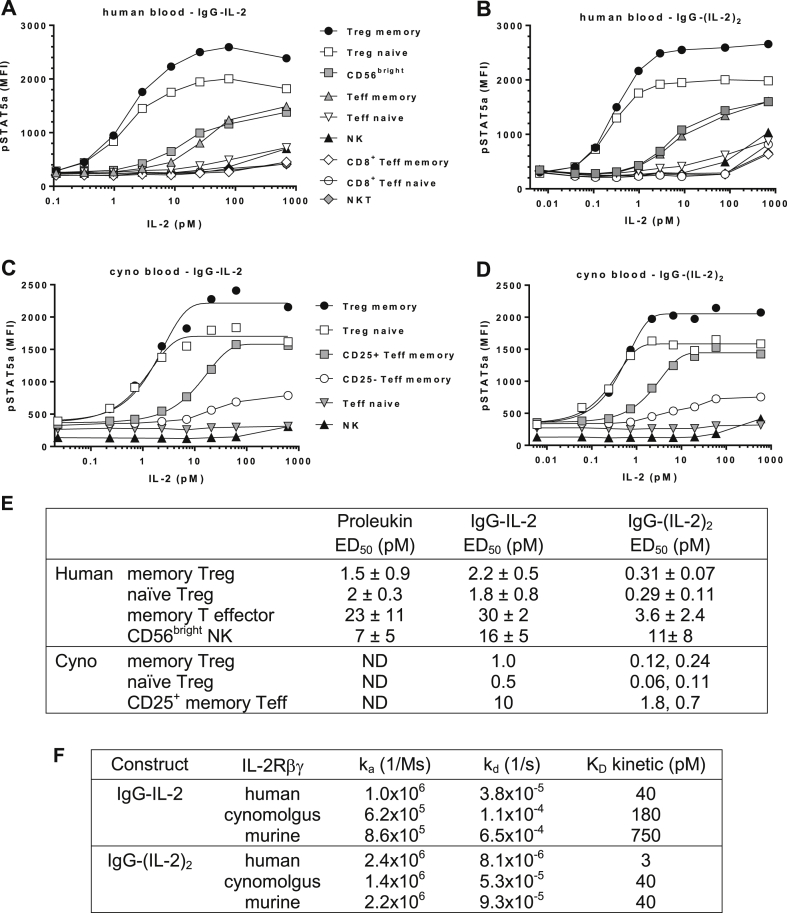
Characterization of IgG-IL-2 and IgG-(IL-2)_2_. (A–D) IgG-IL-2 and IgG-(IL-2)_2_ were tested for their ability to stimulate pSTAT5a in human whole blood and in cynomolgus whole blood; representative human and cynomolgus samples are shown. (E) ED_50_ values for pSTAT5a induction were determined for IgG-IL-2, IgG-(IL-2)_2_ and Proleukin (see Supplemental Fig. 1C for the titration of Proleukin); human blood IgG-IL-2 (*n* = 4 donors, mean ± SD); IgG-(IL-2)_2_ (*n* = 11 donors, mean ± SD); Proleukin (*n* = 3 donors, mean ± SD); cyno blood IgG-IL-2 (*n* = 1), IgG-(IL-2)_2_ (*n* = 2, both determinations shown); ND, not determined. (F) Association and dissociation rate constants and binding affinities of IgG-IL-2 and IgG-(IL-2)_2_ to IL-2Rβγ were determined by Biacore surface plasmon resonance and kinetic analysis.

In human whole blood the induction of pSTAT5a by monovalent forms of IL-2, *i.e.* Proleukin and IgG-IL-2, was comparable across a variety of cell types (Fig. 1A, E, Supplemental Fig. 1C). Human memory and naïve Treg dose responses with IgG-IL-2 produced ED_50_s averaging 1.8 and 2.2 pM and Proleukin ED_50_s averaged 1.5 and 2.0 pM, respectively. Cynomolgus memory and naïve Treg dose responses with IgG-IL-2 in whole blood generated similar ED_50_s to human Tregs, 1.0 and 0.5 pM, respectively (Fig. 1C, E). In contrast, IgG-(IL-2)_2_ dose responses and ED_50_ values improved beyond the 2-fold increase in stoichiometry indicating that the structure of the IgG-(IL-2)_2_ fusion protein enables an increased avidity for the IL-2 receptor. Human Treg ED_50_s for IgG-(IL-2)_2_ averaged 0.3 pM, a 7-fold increase in potency compared to IgG-IL-2 and cynomolgus Treg ED_50_s averaged 0.1 pM, a similar 7-fold increase in potency as human Tregs (Fig. 1B, D, E).

Compared to Tregs, human memory CD4^+^ Teff required 10-fold higher concentrations of all three IL-2 molecules to reach their ED_50_s for IL-2 stimulated pSTAT5 responses (Fig. 1A, B, E, Supplemental Fig. 1C). Although >80% of human CD4^+^ memory cells are CD25^+^, we previously observed reduced pSTAT5 responses to IL-2 in the small proportion of memory cells lacking or having very low CD25 expression [16]. Therefore we analyzed CD25 levels on cynomolgus memory CD4^+^ Teff and found that on average only ∼20% are CD25^+^ (Supplemental Fig. 2E) initiating an assessment of pSTAT5a separately in CD25^+^ and CD25^−^CD4^+^ memory Teff cells. Consistent with their expression of CD25, cynomolgus CD25^+^ memory Teff cells responded to lower concentrations of IL-2 and attained a higher level of pSTAT5a compared to the CD25^−^CD4^+^ memory population (Fig. 1C, D). The ED_50_s for IgG-IL-2 and IgG-(IL-2)_2_ in cynomolgus CD25^+^ memory Teff cells were similar to those seen on human memory Teff cells and approximately 10-fold more of the fusion proteins were required to stimulate a half-maximal pSTAT5 response compared to Tregs (Fig. 1E).

In one additional population of cells, a subpopulation of human NK cells termed CD56^bright^, IL-2 molecules at relatively low concentrations (7–16 pM) induced pSTAT5a (Fig. 1A, B, E, Supplemental Fig. 1C). We did not observe a similar subpopulation of NK cells in cynomolgus that was sensitive to low concentrations of IL-2. The bulk NK population in both human and cynomolgus required 100–1000 pM IL-2 to induce detectable levels of pSTAT5a, as did all other cell types examined (Fig. 1A–D).

The binding affinities of human IgG-IL-2 and IgG-(IL-2)_2_ for human, cynomolgus and murine IL-2Rα and IL-2Rβγ were determined by Biacore surface plasmon resonance (Supplemental Figs. 3 and 4). For all three species of IL-2Rα, the binding of IgG-IL-2 and IgG-(IL-2)_2_ was relatively low affinity, in nM ranges (Supplemental Fig. 3). By contrast, binding to the IL-2Rβγ heterodimer was of very high affinity, in pM ranges. With IgG-IL-2 the *K*_D_ for human, cynomolgus and mouse IL-2Rβγ were 40, 180 and 750 pM, respectively (Fig. 1F and Supplemental Fig. 3). With IgG-(IL-2)_2_, substantial increases in binding affinity were seen to IL-2Rβγ with *K*_D_ values of 3, 40 and 40 pM for human, cynomolgus and murine receptors, respectively. Changing the stoichiometry to IgG-(IL-2)_2_ increased its avidity and produced a 13-fold increase in binding affinity to human IL-2Rβγ, from 40 pM to 3 pM.

### 3.2 Increased Treg frequency and number in cynomolgus after treatment with IL-2 fusion proteins

In our colony (*n* = 51) cynomolgus Tregs averaged 57/mm^3^ blood (median of 56.5/mm^3^, mean of 57.3/mm^3^ ± SD 20.8, ranging from a low of 17/mm^3^ to a high of 96/mm^3^) (Supplemental Fig. 5A) and averaged 4.0% of the total CD4^+^ T cells (median of 4.0%, mean of 4.1% ± SD 1.0, ranging from a low of 2.0 to a high of 6.2) (Supplemental Fig. 5B). Given these ranges in the normal distributions in both Tregs/mm^3^ and Tregs as % of CD4^+^ T cells, it was interesting to find there was no correlation from animal-to-animal between the numbers of Tregs/mm^3^ and the amount of Tregs as the % of CD4^+^ T cells (Supplemental Fig. 5C). Because of this, we present changes in Tregs using both metrics.

**Fig. 2:**
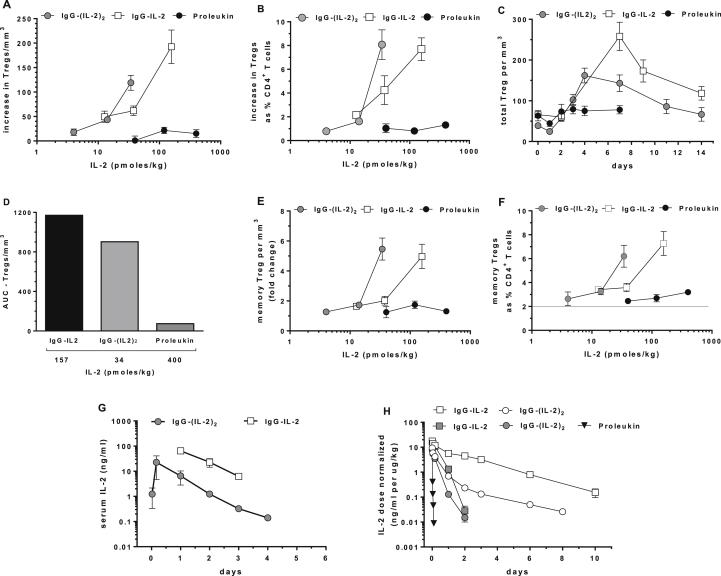
Increases in Tregs after single dose *in vivo* IL-2 treatments. (A) Dose-dependent changes in Tregs are shown as maximal increases in Tregs/mm^3^ blood above individual baselines and (B) as % of total CD4^+^ T cells above individual baselines; baseline Tregs averaged 57/mm^3^ and 4% of total CD4^+^ T cells. (C) Changes in Tregs/mm^3^ over two weeks are shown for Proleukin (400 pmol/kg, *n* = 3), IgG-IL-2 (157 pmol/kg, *n* = 6) and IgG-(IL-2)_2_ (34 pmol/kg, *n* = 5). (D) AUC results for the time-dependent responses in Tregs/mm^3^ are compared. (E) Dose-dependent increases in memory Tregs/mm^3^ are shown as fold-changes and in (F) as % of total CD4^+^ T cells; baseline memory Tregs averaged 2%. (G) Serum levels of IL-2 are shown after SQ dosing of IgG-IL-2 (226 pmol/kg, *n* = 2) and IgG-(IL-2)_2_ (34 pmol/kg, *n* = 2). (H) Dose-normalized serum levels of IgG-IL-2 (600 pmol/kg) and IgG-(IL-2)_2_ (1700 pmol/kg) after IV dosing are shown from NOD.*scid Il2ra*^*null*^ mice (open symbols) and NOD.*scid* mice (shaded symbols) (*n* = 3); for mice dosed with Proleukin (6500 pmol/kg), results from both strains were identical and the pooled results are shown (*n* = 6).

To assess the ability of Proleukin, IgG-IL-2 and IgG-(IL-2)_2_ to expand cynomolgus Tregs *in vivo*, single SQ injections were given and cell subsets in blood monitored over time; all of the results are presented as pmol/kg to facilitate comparisons between the different IL-2 molecules. Treatment-related changes in Tregs were dose-dependent and changes from each animal's individual baseline are shown as the increase in the number of Tregs/mm^3^ blood (Fig. 2A) and as the increase in Tregs as the % of CD4^+^ T cells (Fig. 2B) with “0” equal to no treatment-related change. Following 13, 38 and 157 pmol/kg of IgG-IL-2, cynomolgus Tregs increased both in number: 50/mm^3^, 62/mm^3^ and 193/mm^3^ and as the % of CD4^+^ T cells: 2.1%, 4.2% and 7.7%. After 4, 11 and 34 pmol/kg of IgG-(IL-2)_2_, cynomolgus Treg increases were 18/mm^3^, 44/mm^3^, and 120/mm^3^ and 0.8%, 1.6% and 8.1% of CD4^+^ T cells. Proleukin-induced changes in Tregs with 40, 120 and 400 pmol/kg were 1/mm^3^, 22/mm^3^ and 15/mm^3^ and 1%, 0.8% and 1.3% of CD4^+^ T cells.

After IgG-IL-2 and IgG-(IL-2)_2_ dosing, increases in cynomolgus Tregs peaked between 4 and 7 days with Tregs still above baseline at 14 days, 82% and 69% respectively (Fig. 2C). To quantify the changes in Tregs in Fig. 2C we measured the area under the curve (AUC) for Proleukin and the IL-2 fusion proteins (Fig. 2D). The AUCs for Tregs/mm^3^ were similar between the IL-2 fusion proteins and both were strikingly different from Proleukin: IgG-(IL-2)_2_ yielded an AUC that was 80% of that induced by IgG-IL-2, while Proleukin generated an AUC that was 6% of IgG-IL-2 and 8% of IgG-(IL-2)_2_. As a translational comparison, the increases in the number of cynomolgus Tregs/mm^3^ induced by the low-dose IL-2 fusion proteins (Fig. 2C) are equivalent to the Tregs/mm^3^ in human GVHD patients treated daily with 1 × 10^6^ IU/m^2^ Proleukin [20]. Overall, the dose-dependent increases of cynomolgus Tregs/mm^3^ blood, the increased Treg % in CD4^+^ T cells and the AUCs of the Treg responses over two weeks demonstrate the effectiveness of the IL-2 fusion proteins. These observations support the hypothesis that extending the *in vivo* half-life of IL-2 causes long-lasting induction of Tregs at ultra-low doses.

We monitored for changes in cynomolgus CD4^+^ memory Teff cells and hence potential adverse effects of therapy with a long-lived IL-2 molecule. Absolute numbers of CD4^+^ memory Teffs/ml of blood showed no treatment-related increases following the highest doses of Proleukin, IgG-IL-2 and IgG-(IL-2)_2_ (Supplemental Fig. 1D). There were no detectable increases seen in CD3^−^CD8^+^CD122^+^ NK cells and CD3^+^CD8^+^ T cells. Interestingly a transient drop in lymphocytes and NK cells was seen after all but the lowest doses of Proleukin and IL-2 fusion proteins (Supplemental Fig. 6A, B). The drop in lymphocyte and NK cells occurred within one day of dosing and returned to normal 4–7 days later.

Memory Tregs comprise a majority of cynomolgus Tregs (Supplemental Fig. 5B, D) and both IgG-IL-2 and IgG-(IL-2)_2_ stimulated 5-fold increases in memory Tregs/mm^3^ blood (Fig. 2E) and increased their frequency from 2% to 6–7% of CD4^+^ T cells at the highest doses tested (Fig. 2F); in comparison Proleukin increased their frequency from 2.2% to 3.2%. Increases of naïve Tregs while less pronounced than memory Tregs were seen for all treatments especially at higher doses (Supplemental Fig. 7).

The PK properties of IgG-IL-2 and IgG-(IL-2)_2_ in cynomolgus were compared to the known short duration of Proleukin (IV *t*_1/2_ 13 min, SQ *t*_1/2_ 3.3 h) [30]. IgG-IL-2 and IgG-(IL-2)_2_ had IV *t*_1/2_ of 8 h and SQ *t*_1/2_ of 14 h (Fig. 2G). While prolonged compared to Proleukin, the half-lives were less than the expected 14 day *t*_1/2_ of human IgG1 in cynomolgus [31] suggesting IL-2 receptor-mediated clearance. We tested this by measuring the PK of IgG-IL-2, IgG-(IL-2)_2_ and Proleukin in CD25 knockout (*Il2ra*^*null*^) versus CD25-replete mice (Fig. 2H). Since CD25 knockout mice develop lethal autoimmunity and massive expansion of T cells [32], we developed homozygous CD25 knockout mice lacking an adaptive immune system by virtue of having a *scid* mutation, *i.e.* NOD.*scid IL2ra*^*null*^. Clearance of IgG-IL-2 and IgG-(IL-2)_2_ in CD25-replete NOD.*scid* mice was more rapid and increased 5-fold compared to NOD.*scid IL2ra*^*null*^ mice consistent with CD25 expression and IL-2 uptake by cells outside the adaptive immune system, including endothelial cells, dendritic cells and NK cells [33,34]. With its rapid clearance by the kidneys, levels of Proleukin were below the limit of detection after 2.5 h and no difference was seen in Proleukin clearance when comparing sera from NOD.*scid* and NOD.*scid IL2ra*^*null*^ mice.

### 3.3 STAT5a phosphorylation is prolonged in cynomolgus with IL-2 fusion proteins

**Fig. 3:**
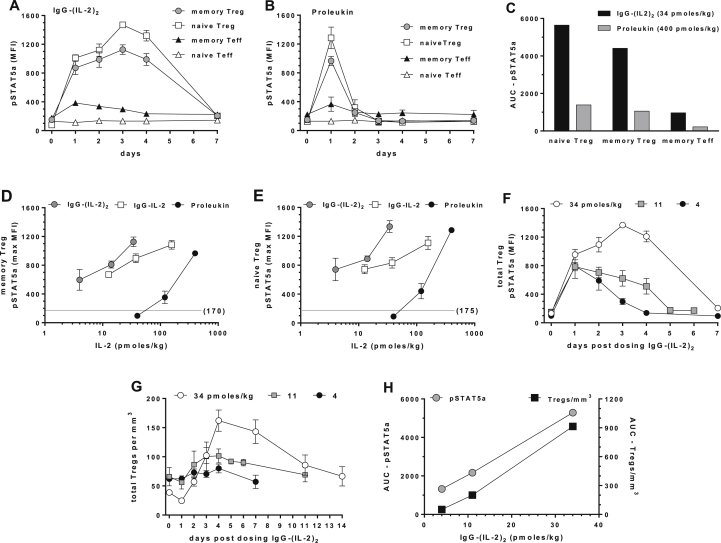
Long-lived pSTAT5a activation after *in vivo* IL-2 treatment. As a measure of IL-2 treatment, pSTAT5a was monitored over one week and whole blood assayed daily without further stimulation. Time courses are shown for (A) IgG-(IL-2)_2_ (34 pmol/kg) and (B) Proleukin (400 pmol/kg). (C) The AUC of pSTAT5a responses are compared for the IgG-(IL-2)_2_ (34 pmol/kg) and Proleukin (400 pmol/kg) time courses. (D) The dose-dependent increases in pSTAT5a are shown for memory Tregs and (E) for naïve Tregs; unstimulated pSTAT5a MFIs were 170 and 175 as indicated. (F) The IgG-(IL-2)_2_ dose-dependent (pmol/kg) time courses for pSTAT5a induction in Tregs and (G) the corresponding increases in Tregs/mm^3^ blood. (H) The dose-dependent responses to IgG-(IL-2)_2_ are expressed as the AUCs for pSTAT5a induction and the corresponding AUCs for increases in Tregs/mm^3^. Group sizes were: IgG-IL-2 (all doses, *n* = 6); IgG-(IL-2)_2_ (11 and 34 pmol/kg, *n* = 5; 4 pmol/kg, *n* = 3); and Proleukin (40 and 400 pmol/kg, *n* = 3; 120 pmol/kg, *n* = 4).

IL-2 stimulation of pSTAT5a is a sensitive proximal biomarker of Treg activation. To assess the magnitude and duration of activation after *in vivo* treatment, we monitored pSTAT5a over time by sequentially collecting blood after treatment without further *ex vivo* stimulation. Clear kinetic differences in memory and naïve Treg pSTAT5a responses were seen between the highest doses of IgG-(IL-2)_2_ (34 pmol/kg) and Proleukin (400 pmol/kg): maximal Treg pSTAT5a levels were maintained for 4 days with IgG-(IL-2)_2_ (Fig. 3A) and for one day with Proleukin (Fig. 3B). AUC analysis quantified Proleukin-induced naïve and memory Treg responses at 25% of the IgG-(IL-2)_2_ responses (Fig. 3C). Both IgG-(IL-2)_2_ and Proleukin stimulated pSTAT5a in CD4^+^ memory Teff cells but not to near the extent of Tregs and is reflected by the lower maximum MFIs and AUCs. CD25^−^CD4^+^ naïve Teff cells were unresponsive using the criteria of pSTAT5a induction and had no quantifiable AUC.

Proleukin, IgG-IL-2 and IgG-(IL-2)_2_ all stimulated dose-dependent pSTAT5a responses in memory and naïve Tregs (Fig. 3D, E). Proleukin stimulated maximal pSTAT5a responses at 400 pmol/kg, had limited effects at 120 pmol/kg and had no effect at 40 pmol/kg. In contrast, IgG-IL-2 and IgG-(IL-2)_2_ stimulated pSTAT5a at the lowest administered doses, 4 and 13 pmol/kg, respectively and both IgG-IL-2 and IgG-(IL-2)_2_ showed dose-dependent superiority at stimulating maximal and sustained *in vivo* pSTAT5a responses.

We next compared IgG-(IL-2)_2_ dose and time-dependent pSTAT5a induction in Tregs (Fig. 3F) with their *in vivo* expansion (Fig. 3G). The extent of Treg expansion was dependent and correlated with a pSTAT5a response that was sustained at a sufficient level for an extended signaling period. The highest dose of IgG-(IL-2)_2_ (34 pmol/kg) stimulated Treg pSTAT5a that was maintained for four days and as a consequence, Treg numbers increased from 38 to 162/mm^3^ and were still elevated on days 11 (126%) and 14 (76%). The lower doses of IgG-(IL-2)_2_ (11 and 4 pmol/kg) stimulated less pSTAT5a and smaller increases in the number of Tregs/mm^3^. These dose and time-dependent effects were quantified as AUCs and then compared (Fig. 3H). A clear correlation and dose relationship was seen between the AUCs for pSTAT5 signaling and the AUCs for increased Tregs/mm^3^. The results ranged from a threshold of effects at the lowest dose to maximal changes at the highest dose.

### 3.4 CD25, FOXP3, Ki-67 and soluble CD25 are biomarkers of IL-2 treatment in cynomolgus

**Fig. 4:**
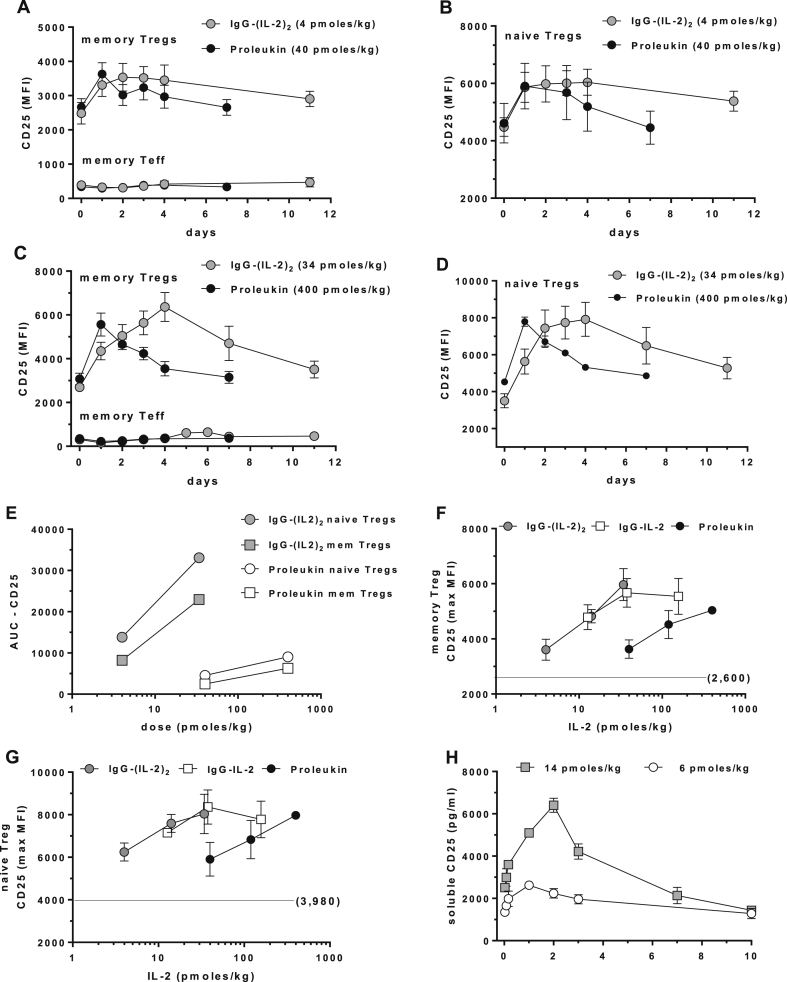
Increases in CD25 as a biomarker of IL-2-induced *in vivo* activation. Cell surface CD25 on memory Tregs and Teff and naïve Tregs are shown following (A, B) the lowest doses of IgG-(IL-2)_2_ (4 pmol/kg) and Proleukin (40 pmol/kg) and (C, D) after the highest doses of IgG-(IL-2)_2_ (34 pmol/kg) and Proleukin (400 pmol/kg). (E) The AUC results for CD25 stimulation are shown for low and high-dose IgG-(IL-2)_2_ and Proleukin on naïve and memory Tregs. (F, G) The dose-dependent increases in CD25 for IgG-IL-2, IgG-(IL-2)_2_ and Proleukin are shown for memory Tregs and naïve Tregs; unstimulated CD25 MFIs were 2600 and 3980 as indicated. (H) Soluble CD25 was measured in serum from IV studies with IgG-(IL-2)_2_. Group sizes were: IgG-IL-2 (all doses, *n* = 6); IgG-(IL-2)_2_ (11 and 34 pmol/kg, *n* = 5; 4 pmol/kg, *n* = 3); and Proleukin (40 and 400 pmol/kg, *n* = 3; 120 pmol/kg, *n* = 4).

We measured dose and time-dependent increases in CD25 expression on Tregs and found upregulation of CD25 was more long-lived than pSTAT5a. At ultra-low doses of IgG-(IL-2)_2_, maximal CD25 responses were maintained in memory (Fig. 4A, C) and naïve Tregs (Fig. 4B, D) out to 4 days and were still above background at 11 days. Proleukin stimulated CD25 responses at 24 h in memory (Fig. 4A, C) and naïve Tregs (Fig. 4B, D) and returned to normal at 7 days. We quantified AUCs for Treg CD25 responses and compared them to each other (Fig. 4E); this illustrates that increasing the half-life of IL-2 via the fusion proteins had a major impact on the breadth and depth of *in vivo* activation. No changes in the expression of CD25 were seen in CD25^+^CD4^+^ memory Teff cells in any experiment (Fig. 4A, C). IL-2 treatment in man is known to increase levels of circulating sCD25 [35] and we observed a dose-dependent increase of sCD25 in cynomolgus after receiving IgG-(IL-2)_2_ (Fig. 4H).

**Fig. 5:**
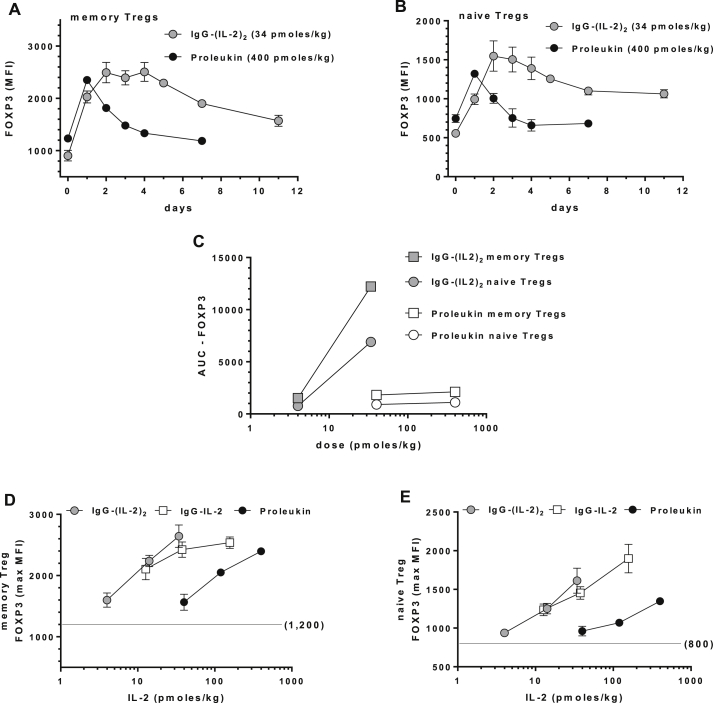
Intracellular FOXP3 as a biomarker of IL-2-induced *in vivo* activation. (A, B) The time-dependent ability to stimulate FOXP3 is shown for the highest doses of IgG-(IL-2)_2_ (34 pmol/kg) and Proleukin (400 pmol/kg) in memory Tregs and naïve Tregs. (C) The AUC results for FOXP3 stimulation are shown for low and high-dose IgG-(IL-2)_2_ and Proleukin in memory and naïve Tregs. (D, E) The dose-dependent increases in FOXP3 for IgG-IL-2, IgG-(IL-2)_2_ and Proleukin are shown for memory Tregs and naïve Tregs; unstimulated FOXP3 MFIs were 1200 and 800 as indicated. Group sizes were: IgG-IL-2 (all doses, *n* = 6); IgG-(IL-2)_2_ (11 and 34 pmol/kg, *n* = 5; 4 pmol/kg, *n* = 3); and Proleukin (40 and 400 pmol/kg, *n* = 3; 120 pmol/kg, *n* = 4).

Expression of FOXP3 is a hallmark of Tregs and was upregulated in cynomolgus memory and naïve Tregs as a consequence of *in vivo* activation and as we found for CD25, its expression was longer-lived than pSTAT5a responses. The lowest doses of Proleukin and IgG-(IL-2)_2_ stimulated small increases in FOXP3 that quickly returned to normal (not shown) while higher doses induced greater and more sustained effects (Fig. 5A, B). Similar to CD25, IgG-(IL-2)_2_ (34 pmol/kg) stimulated Treg FOXP3 expression for 4–5 days and remained above baseline at 11 days. Proleukin responses (400 pmol/kg) peaked at 24 h and returned to normal by days 4–7. As with CD25, we quantified AUCs for the Treg FOXP3 responses and compared them to each other (Fig. 5C), again showing the major impact on *in vivo* activation the fusion proteins generated in memory and naïve Tregs, *i.e.* a 6-fold larger AUC at a 10-fold lower dose compared to Proleukin. There was no induction of FOXP3 in CD4^+^ memory Teff cells (not shown).

**Fig. 6:**
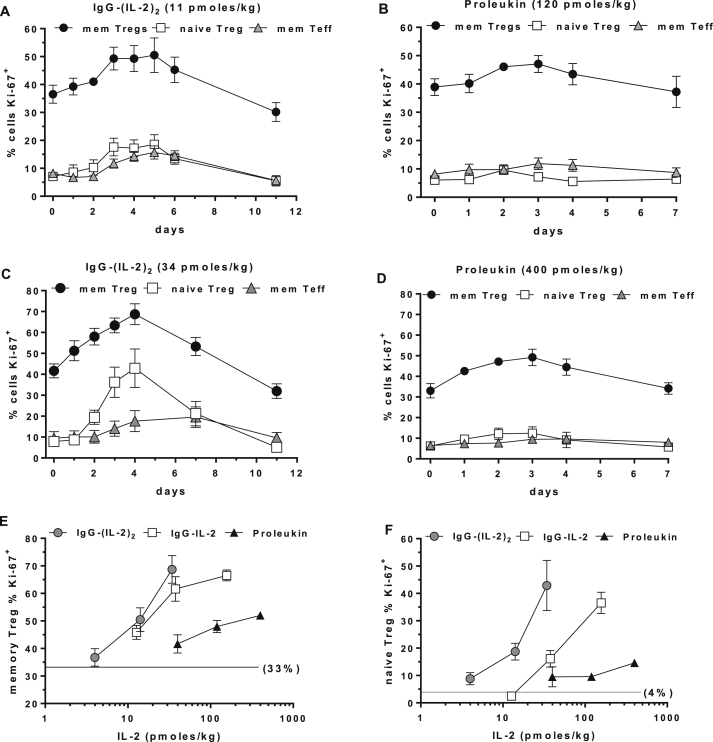
Intracellular Ki-67 as a marker for induction into cell cycle and proliferation. The ability to stimulate proliferation was quantified using Ki-67 as a biomarker of IL-2-induced *in vivo* activation. (A, C) The ability to stimulate Ki-67 is shown for low and high doses of IgG-(IL-2)_2_ and (B, D) compared to low and high doses of Proleukin. (E, F) Dose-dependent increases in Ki-67 for IgG-IL-2, IgG-(IL-2)_2_ and Proleukin are shown for memory Tregs and naïve Tregs; the unstimulated % Ki-67^+^ memory and naïve Tregs were 33% and 4%, respectively, as indicated. Group sizes were: IgG-IL-2 (all doses, *n* = 6); IgG-(IL-2)_2_ (11 and 34 pmol/kg, *n* = 5; 4 pmol/kg, *n* = 3); and Proleukin (40 and 400 pmol/kg, *n* = 3; 120 pmol/kg, *n* = 4).

Ki-67 is an intracellular marker of entry into cell cycle and proliferation. Untreated memory Tregs had the highest basal level of proliferation, averaging 33% Ki-67^+^, while naïve Tregs and CD4^+^ memory Teff averaged 4% and 8% cells in cycle, respectively. After ultra-low dose IgG-(IL-2)_2_ treatment the number of memory Tregs, naïve Tregs and memory Teff cells in cell cycle increased 14%, 12% and 9% (Fig. 6A) and after Proleukin 8%, 4% and 4% (Fig. 6B). Following the highest dose of IgG-(IL-2)_2_, memory Tregs, naïve Tregs and memory Teff cells in cell cycle increased by 36%, 47% and 14% (Fig. 6C) and after Proleukin by 16%, 6% and 3% (Fig. 6D). Maximal increases in Ki-67^+^ cells peaked 5 days after IgG-(IL-2)_2_ and 3 days after Proleukin. Treatment-related increases in Ki-67^+^ cells for memory and naïve Tregs are shown as maximum dose-dependent effects of IgG-IL-2, IgG-(IL-2)_2_ and Proleukin (Fig. 6E, F) with IgG-(IL-2)_2_ having a >10-fold advantage in potency.

### 3.5 FOXP3 and CTLA4 remain demethylated in cynomolgus Tregs after IL-2 induced in vivo expansion

Constitutive expression of FOXP3 in mouse Tregs is associated with functional suppression and relies on demethylation of CpG DNA methylation sites in the Treg-specific demethylated region (TSDR) within intron 1 of *Foxp3* [36]. An orthologous region in human *FOXP3* is also specifically demethylated in human Tregs. Human Teff cells activated and differentiated *in vitro* express FOXP3 protein but unlike Tregs, the TSDR remains methylated [37,38]. Similarly, mouse Tregs are preferentially demethylated within a region of *Ctla4* exon 2 compared to conventional T cells [39].

**Fig. 7:**
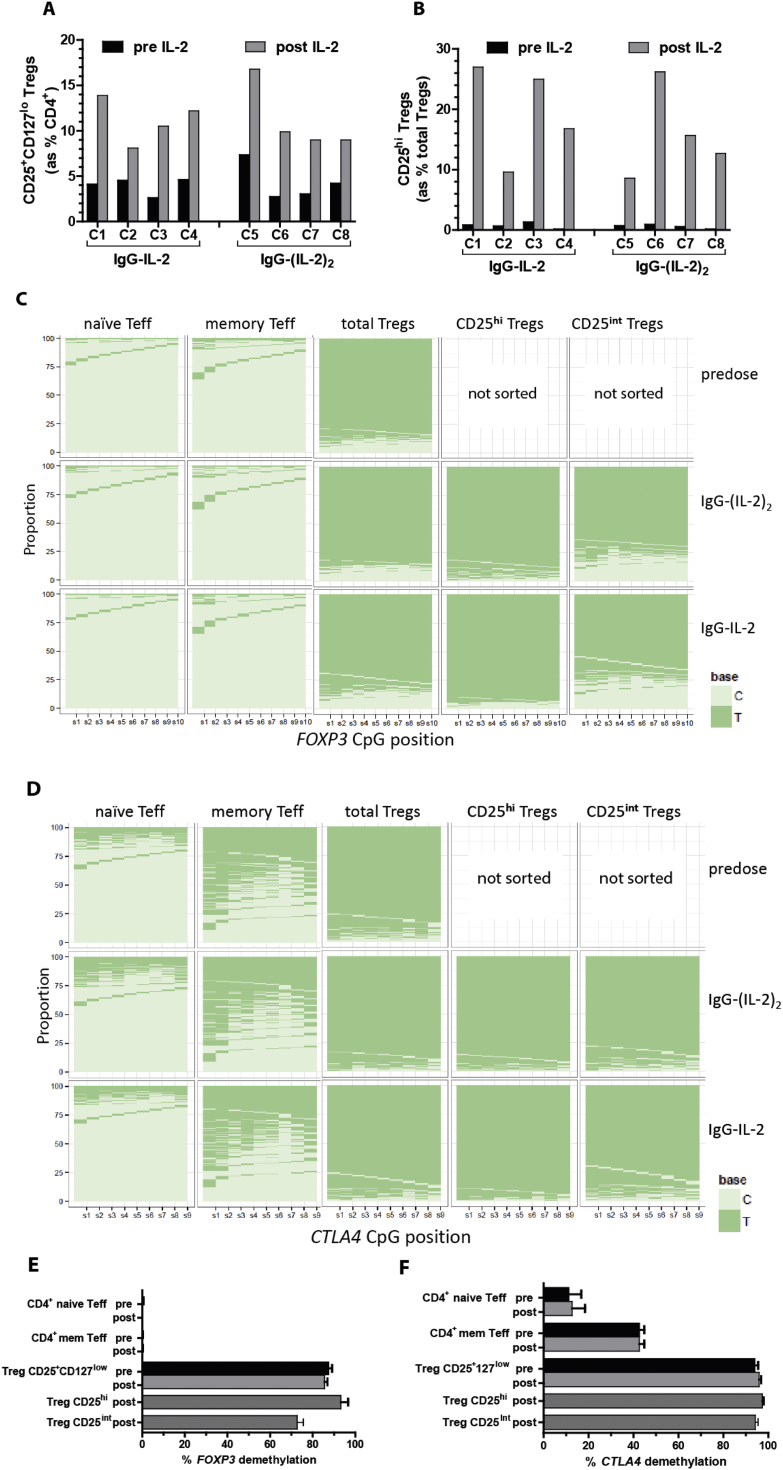
DNA demethylation before and after IL-2 treatment. The epigenetic signatures of *FOXP3* and *CTLA4* were measured before and after IgG-IL-2 (157 pmol/kg, *n* = 4, C1–C4) or IgG-(IL-2)_2_ (34 pmol/kg, *n* = 4, C5–C8) induced *in vivo* expansion. (A, B) Baseline and post-treatment induction of Tregs and CD25^hi^ Tregs are shown. The methylation status of sorted naïve and memory Teff and total, CD25^inter^ and CD25^hi^ Tregs (Supplemental Fig. 8) was assessed with Next Generation Sequencing; methylated cytosines are read as C and demethylated cytosines as T. (C, D) To visualize the demethylation results, the proportion of cytosines methylated versus demethylated at each of the relevant CpG sites is shown for *FOXP3* and *CTLA4*. (E, F) These demethylation results are graphically shown for *FOXP3* and *CTLA4*.

To determine if cynomolgus Tregs retained epigenetic signatures consistent with being functional Tregs after *in vivo* expansion, we developed NGS-based sequencing of the regions in *FOXP3* and *CTLA4* homologous to those defined in mice and humans that are preferentially demethylated in Tregs [39,40]. We assessed methylation in sorted cynomolgus Treg and non-Treg CD4^+^ T cell subsets before and after dosing with IgG-IL-2 and IgG-(IL-2)_2_ (Fig. 7). The doses chosen to expand Tregs were based on the previous tests where IgG-(IL-2)_2_ (34 pmol/kg) increased Tregs from 4% to 11% and IgG-IL-2 (157 pmol/kg) increased Tregs from 4% to 12%. Using these doses again, IgG-IL-2 and IgG-(IL-2)_2_ each raised total Tregs from 4% of total CD4^+^ cells to 11.2% (Fig. 7A). Only a small number of memory Tregs (∼300 cells/ml of blood) expressed high levels of CD25 (Supplemental Fig. 7A, B) and are considered “activated” Tregs [40]. While blood volume limitations prevented assessing this rare subset before treatment, the activated Treg population greatly expanded after treatment (Supplemental Fig. 7C, D) and allowed them to be sorted for epigenetic analysis (Supplemental Fig. 7F, G). The CD25^hi^ Tregs increased 30-fold from 0.5 to 0.7% of total Tregs to 20% and 16% of total Tregs after IgG-IL-2 and IgG-(IL-2)_2_, respectively (Fig. 7B). A human memory Treg subset with intermediate levels of CD25 (CD25^int^) has been shown to contain a high proportion of cells (60%) lacking the epigenetic signature of Tregs [40]. Following expansion there were a sufficient number of CD25^int^ Tregs to isolate for analysis (Supplemental Fig. 7E).

Bisulfite sequencing of DNA from cells sorted from pre-dose blood confirmed the Treg-specificity of the cynomolgus *FOXP3* TSDR (Fig. 7C, E): total Tregs for the eight cynomolgus in the test were on average 85% demethylated whereas memory and naïve Teff (Supplemental Fig. 7H–J) were both <1% demethylated. Although *CTLA4* demethylation in exon 2 was not completely specific for Tregs, as reported for mice [39], Treg-biased demethylation was observed prior to expansion with Tregs > memory Teff ≫ naïve Teff (Fig. 7D, F). Notably, after undergoing significant *in vivo* expansion, Tregs retained the identical *FOXP3* and *CTLA4* methylation patterns compared to pre-dose Tregs indicating that they retained their functional signatures (Fig. 7C, D). Importantly, total CD25^+^CD127^lo^ Tregs as well as CD25^hi^ activated Tregs and CD25^int^ Tregs retained a Treg demethylation pattern indicating that the Treg expansion following IL-2 dosing was not attributable to highly activated effector CD4^+^ T cells.

### 3.6 Multiple dosing with Proleukin expands cynomolgus Tregs

**Fig. 8:**
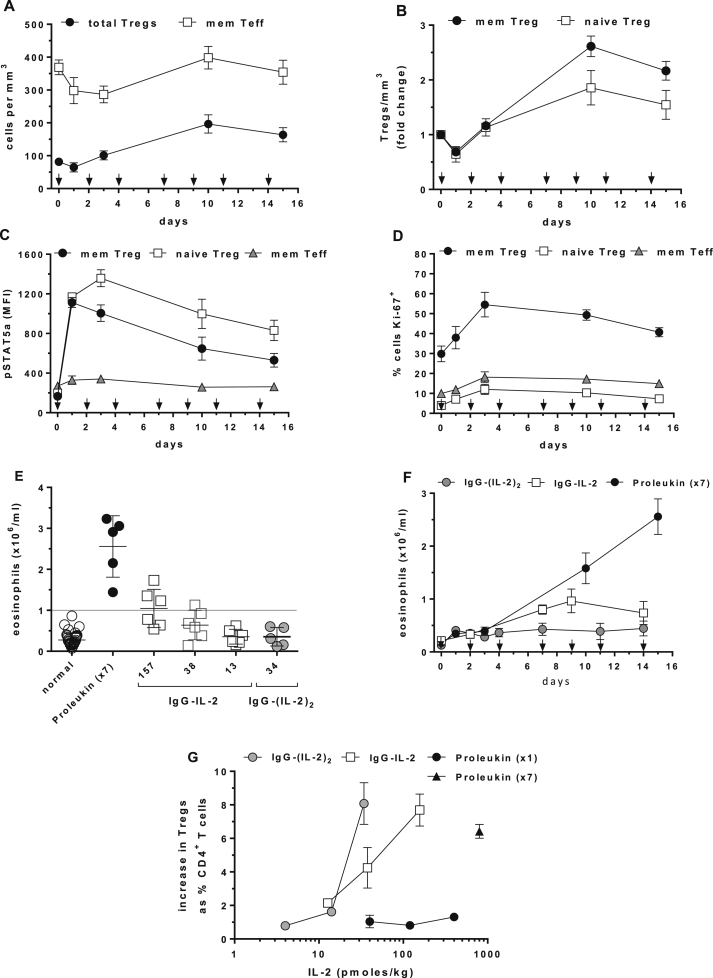
Effects of multiple dose Proleukin and single dose IgG-IL-2. (A, B) Proleukin was given every other day (arrows) at 800 pmol/kg and changes in Tregs/mm^3^ and memory Teff/mm^3^ were assessed as were fold-changes in memory and naïve Tregs. (C, D) pSTAT5a activation in memory and naive Tregs and memory Teff are shown as well as % Ki-67^+^ cells for induction into cell cycle. (E) Maximal eosinophil counts are shown for multi-dose Proleukin and IL-2 fusion proteins. (F) The time course of eosinophilia for multi-dose Proleukin and single-dose IgG-IL-2 (157 pmol/kg) or IgG-(IL-2)_2_ (34 pmol/kg). (G) Comparison of single and multiple-dose Proleukin with single-dose IgG-IL-2 and IgG-(IL-2)_2_ for stimulating Tregs are shown as increases above individual baselines as % of CD4^+^ T cells. Group sizes were: multi-dose Proleukin (*n* = 5); IgG-IL-2 (all doses, *n* = 6); IgG-(IL-2)_2_ (11 and 34 pmol/kg, *n* = 5; 4 pmol/kg, *n* = 3); and Proleukin (40 and 400 pmol/kg, *n* = 3; 120 pmol/kg, *n* = 4).

In this study, single doses of Proleukin equivalent to 1.2 × 10^6^ IU/m^2^ in humans (Supplemental Fig. 1D) stimulated modest 30% increases in Tregs (Fig. 2). In contrast, Tregs in type 1 diabetics increased 100–200% (2–3-fold) following 4.5 × 10^6^ IU Proleukin/per person three times per week for four weeks [35,41]. Using body surface area for dose translation across species [42], 4.5 × 10^6^ IU/person converts to an equivalent cynomolgus dose of 200,000 IU/kg (800 pmol/kg); hence we dosed five cynomolgus with Proleukin at 800 pmol/kg three times per week for 15 days for a total of seven doses. With this multi-dose regimen, cynomolgus Tregs increased from 81 to 197/mm^3^ (2.4-fold, 143%) (Fig. 8A) and memory and naïve Tregs increased 2.6-fold and 1.9-fold, respectively (Fig. 8B), results equal to T1D patients receiving dose equivalent treatment [35]. The number of Tregs appeared to decline with continued dosing suggesting enhanced migration or exhaustion. Despite continued dosing, pSTAT5a was maximal for only 1–3 days and declined thereafter (Fig. 8C). Compared to single-dose Proleukin (Fig. 6D), multiple-dosing did not increase further the number of Ki-67^+^ Tregs in cell cycle but did maintain their numbers for the duration of dosing (Fig. 8D). Treg CD25 levels increased on days 1–3 followed by a decline in expression (not shown). As in other experiments, there was a transient lymphopenia (Supplemental Fig. 6A) and there were no increases in memory Teff (Fig. 8A), naïve Teff (not shown) or NK cells (Supplemental Fig. 6B).

### 3.7 IL-2-induced eosinophilia is dose dependent

In humans, repeated doses of 1 × 10^6^ to 4.5 × 10^6^ IU Proleukin induce a significant increase in eosinophils [20,35]. In this study, cynomolgus eosinophils were measured in normal pre-IL-2 treatment bloods (*n* = 44) with a median of 0.23 × 10^6^/ml, a mean of 0.28 × 10^6^/ml ± SD 0.16, with a low of 0.07 × 10^6^/ml and high of 0.86 × 10^6^/ml (Fig. 8E). Using these colony reference values we considered that numbers above 1 × 10^6^/ml qualified as eosinophilia. After applying this criterion, all cynomolgus given multiple-dose Proleukin (800 pmol/kg, 7 doses) developed eosinophilia. IgG-IL-2 had a clear dose-dependent ability to increase eosinophils above 1 × 10^6^/ml while no single doses of IgG-(IL-2)_2_ or Proleukin induced eosinophilia. The increase in eosinophils induced by multiple-dose Proleukin continued throughout the dosing regimen while that of IgG-IL-2 peaked at day 7 and declined (Fig. 8F).

These results in cynomolgus demonstrate that a reduction of the IL-2 dose facilitated by an extended half-life and increased avidity for the IL-2R provided a greater degree of cell specificity in favor of Tregs. To illustrate this in light of the ability to induce eosinophilia (Fig. 8E), we compiled the results for single and multiple-dose Proleukin and single-dose IgG-IL-2 and IgG-(IL-2)_2_ for their ability to increase Tregs (Fig. 8G). Single-dose Proleukin had little effect on Tregs and no effect on eosinophils while multiple-dose Proleukin (×7) substantially increased Tregs and eosinophils in 100% of the animals. IgG-IL-2 at its highest dose stimulated similar increases in Tregs as multi-dose Proleukin but only induced eosinophilia in 50% of the animals. The advantage of a prolonged half-life and increase in avidity was clearest when IgG-(IL-2)_2_ at an ultra-low dose stimulated an increase in Tregs similar to multi-dose Proleukin and IgG-IL-2 in 100% of the animals but had no effect on eosinophils.

## 4 Discussion

Over the last 20 years we have progressed from discovering that IL-2 and IL-2RA are genetically associated with autoimmune diabetes and the functional state of Tregs to seeing dramatic clinical success with IL-2 in chronic GVHD. The central role of IL-2 in the maintenance of self-tolerance and Treg function is now immunological canon and many attempts are being made to harness Tregs to combat a variety of autoimmune and inflammatory diseases. The recent clinical successes with Proleukin are noteworthy since pharmacologically it is a drug with limitations: its short half-life requires daily or every other day injection and the doses used to date stimulate CD4^+^ T effector cells, NK cells and eosinophils in addition to Tregs. Our goal was to develop and characterize IL-2 molecules with improved pharmacologic profiles that could be delivered less frequently and at lower doses than Proleukin and selectively expand Tregs that maintained their epigenetic profiles at *FOXP3* and *CTLA4*.

Increasing the *in vivo* half-life of IL-2 by fusion to IgG1, *i.e.* IgG-IL-2, results in a molecule that can induce a 4-fold increase in Tregs after a single dose in cynomolgus, a response that multiple-dose, but no single dose, of Proleukin can achieve. Increasing the stoichiometry and hence the avidity, *i.e.* IgG-(IL-2)_2_, increases the potency and stimulates a similar increase in Tregs albeit at a 5-fold lower dose than IgG-IL-2. A detailed characterization of the *in vivo* dose responses for Proleukin and IgG-(IL-2)_2_ highlights that the magnitude and duration of Treg expansion, defined by its AUC, correlates with the magnitude and duration of pSTAT5a upregulation, also defined by its AUC. Single doses of Proleukin that increase pSTAT5a for one day have a minimal AUC and as a consequence little impact on Treg numbers; whereas single dose IgG-IL-2 and IgG-(IL-2)_2_ or multiple-dose Proleukin stimulate pSTAT5a that is sustained for 4 days resulting in 3–4-fold larger pSTAT5a AUCs and corresponding increases in Tregs and the AUCs of Treg/mm^3^. Intermediate levels and duration of pSTAT5a induction result in moderate increases in Tregs. Following *in vivo* activation with Proleukin and IgG-(IL-2)_2_, Treg cell surface CD25 as well as intracellular FOXP3 and Ki-67, increased in a dose-dependent manner and persisted longer than the corresponding pSTAT5a responses; the effects of IgG-(IL-2)_2_ were >10-fold more potent and persisted longer than those induced by Proleukin. Of particular significance, the cynomolgus Tregs present after IgG-IL-2 and IgG-(IL-2)_2_-induced *in vivo* expansion retain their fully demethylated *FOXP3* and *CTLA4* epigenetic signatures indicating a functional suppressive phenotype. Although we did not directly test the suppressive capacity of Tregs expanded *in vivo* by IgG-IL-2 and IgG-(IL-2)_2_, Tregs induced to expand by Proleukin have been documented to be functional using *in vitro* suppression assays in both human [20,21] and cynomolgus [43,44] studies.

Activation and expansion of Tregs by IL-2, most likely in combination with T cell receptor-driven signaling [45], seems to require a sufficient magnitude and duration of IL-2 exposure. Although pSTAT5a levels were not reported, Treg expansion in normal human volunteers required daily dosing for five days with Proleukin and was clearly dose dependent [23], a finding that is consistent with the sustained, suprathreshold effects observed in cynomolgus in this study given single doses of IgG-IL-2 and IgG-(IL-2)_2_ or multiple-doses of Proleukin.

The ability of cynomolgus to respond and differentiate amongst different forms and doses of IL-2 with varying degrees of activation and increases in Tregs speaks to their utility as a translational preclinical species. In fact, single doses of IgG-IL-2 and IgG-(IL-2)_2_ replicated the increased number of Tregs (Fig. 2A, B) seen in GVHD patients given daily Proleukin [20]. Furthermore, Proleukin given to cynomolgus following the same multiple-dose protocol at the human equivalent dose (Fig. 8F, G) achieved the same increases in Tregs and eosinophils as patients with type 1 diabetes [35].

The long half-lives of IgG-IL-2 and IgG-(IL-2)_2_ enable the detection of receptor-mediated clearance of IL-2 *in vivo*; the half-lives of the fusion proteins are five times longer in mice in the absence of the high affinity IL-2 receptor (Fig. 2H). The competition for injected IL-2 by different cell populations and the upregulation of IL-2 receptors in response to injections of the cytokine [46] are important considerations when interpreting IL-2 doses required for preferential Treg expansion.

One limitation of the current study is that cynomolgus NK cells do not expand in response to IL-2 as readily as humans. Daily dosing of 1–3 × 10^6^ units of proleukin increases NK cells in patients with chronic GVHD and type 1 diabetes [20,24]. With multiple-dose Proleukin in cynomolgus the expansion of Tregs and eosinophils occurs as expected based on humans treated with the equivalent dosing regimen but not the increase in NK cells [35]. Similar to our study, there was no increase in cynomolgus NK cells with 28 days of daily Proleukin given at levels sufficient to induce a large expansion of Tregs [43]. It is unclear why cynomolgus NK cells do not expand following IL-2 dosing, unlike humans and mice [34,35] especially since the NK cells have high levels of CD122 and can be killed by an IL-2-immunotoxin (denileukin difitox) [47]. The failure of low-dose IL-2 to expand cynomolgus NK cells *in vivo* means that this aspect of IL-2 immunotherapy using novel, long-lived molecules will need to be addressed in future human studies. Despite these differences, the pharmacokinetic and pharmacodynamic analyses in this cynomolgus study strongly support the hypothesis that increasing the half-life of IL-2 allows for lower doses of IL-2 to be delivered far less frequently thereby favoring prolonged Treg-specific cell expansion.

Ultra-low doses of IL-2 have the potential to personalize treatments for different autoimmune and inflammatory diseases as well as individual patients. The current study illustrates that a wide range of dose-dependent sustained Treg activation states can be readily induced by ultra-low dose IgG-(IL-2)_2_, evidenced by the pSTAT5a responses as well as CD25, FOXP3 and Ki-67. With dose selection of a potential therapeutic such as IgG-(IL-2)_2_, Tregs could be activated to varying degrees and individualized to generate the appropriate level of *in vivo* Treg expansion as the clinical circumstances and individual patient responses to treatment dictate.

## 5 Conclusions

Our results in nonhuman primates show that the development of long-lived IL-2 molecules enables the extended *in vivo* delivery of this cytokine that is essential for Treg maintenance and does so in a manner that minimizes the expansion of eosinophils, effector CD4^+^ and CD8^+^ T cells. By increasing the stoichiometry of IL-2 on the IgG molecule, *e.g.* IgG-(IL-2)_2_, we not only enhanced binding to the IL-2 receptor but generated a molecule with sustained and specific effects on Tregs at ultra-low *in vivo* doses. A striking finding facilitated by the *in vivo* dose response to IgG-(IL-2)_2_ was the correlation between the extent of sustained suprathreshold pSTAT5a induction in Tregs and the ability to increase the absolute numbers of Tregs/mm^3^ in blood. These long-lived IL-2 fusion proteins have the potential to improve IL-2 immunotherapy in conditions where Proleukin has already been shown to provide benefit, GVHD and hepatitis C-induced vasculitis [20–22], as well as other inflammatory and autoimmune conditions.

## Authorship contributions

C.J.M.B., Y.S., U.M.N., R.J.H., C.K., L.S.W. and L.B.P. participated in the design and interpretation of the experiments and results. C.J.M.B., Y.S., U.M.N., J.C., S.H., X.Y., R.J.H., A.F-G., L.S.W. and L.B.P. participated in the acquisition and analysis of data. M.L.P. contributed to the design of experimental FACS panels. C.K., P.U., R.J.H., L.S.W., L.B.P. and E.M. designed the immunocytokines and O.A. generated the immunocytokines. I.W. and A.F-G. characterized the immunocytokines *in vitro* and biochemically. C.J.M.B., L.S.W. and L.B.P. wrote the manuscript.

## Funding

This work was supported by Wellcome Trust Grant 091157, JDRF International Grant 9-2011-253, the National Institute for Health Research Cambridge Biomedical Research Centre, and the Medical Research Council Cusrow Wadia Fund. The Cambridge Institute for Medical Research (CIMR) is in receipt of a Wellcome Trust Strategic Award (100140). U.M.N. was the recipient of a Hoffmann-La Roche postdoctoral fellowship.

## Disclosure

L.B.P., L.S.W., R.J.H., C.K., E.M. and P.U. are co-inventors on patents for IL-2 fusion proteins (WO2014/023752A1 and US2014/0044675A1). LBP and YS are former employees of Hoffmann-La Roche. I.W., O.A., E.M., P.U., C.K., R.J.H. and A.F-G. are stakeholders in Hoffmann-La Roche.

## Appendix A Supplementary data

Supplemental Fig. 1 IL-2 fusion protein schematics, Proleukin whole blood activity and *in vivo* dose conversions. (A, B) The human IgG-IL-2 and IgG-(IL-2)_2_ fusion proteins are illustrated: the V-region germline sequences have no known binding to human cells or tissues; the point mutations in the CH2 region of the human IgG1 molecule render the molecule effector-silent; knobs-into-holes technology was used to create monovalent IgG-IL-2. (C) The ability of Proleukin to stimulate pSTAT5a in human whole blood (*n* = 3 donors) is shown. (D) Maximal changes in CD4^+^ memory Teff cells are shown for Proleukin (400 pmol/kg, *n* = 3), IgG-IL-2 (157 pmol/kg, *n* = 6) and IgG-(IL-2)_2_ (34 pmol/kg, *n* = 5). (E) Using body surface area to translate dosage across species, the table shows the doses of Proleukin given to cynomolgus and their equivalent doses in humans (in IU/m^2^ or * per person).

Supplemental Fig. 2 Flow cytometry gating of cynomolgus blood cell subsets. (A) Lymphocytes were isolated and (B) refined into single cells, a step performed during analysis of all tubes. (C) For intracellular staining, CD3^+^ T cells were split into CD4^+^ and CD4^−^CD8^+^ subsets (in the *ex vivo* tube CD4^+^ T cells were gated without CD3). (D) CD3^+^CD4^+^ T cells were separated into memory (CD45RA^−^ and CD45RA^intermediate^) and naïve (CD45RA^+^) subsets. CD45RA^intermediate^ cells were included in the memory gate because analyses of this “CD45RA transitional subset” revealed that molecules such as CD25, FOXP3 and Ki-67 had expression patterns more similar to CD45RA^−^ as compared to CD45RA^+^ CD4^+^ T cells. Cells expressing very high levels of CD45RA were excluded from analysis: they were not present in all animals and showed no response to IL-2. Further investigation of the cells expressing high levels of CD45RA was performed: (M) CD3^+^CD4^+^ T cells were gated and (N) we found that CD8 was co-expressed on these cells. (E, F) CD3^+^CD4^+^ T cells not expressing high levels of levels of CD45RA were separated into Tregs and Teff in memory and naïve subsets for analyses. (E) CD25^+^ memory Teff are also shown. (G, H, I) The pSTAT5a status of total CD4^+^ memory T cells are shown responding *in vitro* to no, 5.7 pM and 570 pM IgG-(IL-2)_2_ demonstrating that the subset of memory cells expressing the highest levels of CD25 require lower concentrations of IL-2 to stimulate pSTAT5a increases. (J) Using the surface panel, CD3^+^CD8^+^ T cells were gated as were CD3^−^CD8^+^ NK cells. (K) The CD3^−^ population is shown, identifying the CD3^−^CD8^+^ NK cells as CD122^+^. (L) The CD3^−^ population is shown in demonstrating that CD8^+^CD3^−^ cells are highest for CD45RA expression, allowing our identification of CD3^−^CD45RA^hi^ cells as NK cells in our pSTAT5a intracellular panel. CD16 was used as a marker in the surface panel but showed heterogenous staining in the CD3^−^CD8^+^CD122^+^ subset across animals and was hence not used as an NK marker (data not shown).

Supplemental Fig. 3 Surface plasmon resonance binding assessment to IL-2Rα. Surface plasmon resonance sensorgrams of (A, C, E) IgG-IL-2 and (B, D, F) IgG-(IL-2)_2_ binding to immobilized (A, B) human, (C, D) cynomolgus, and (E, F) murine IL-2Rα. The receptor was chemically immobilized by amine coupling on a Biacore CM5 sensorchip. To determine the *K*_D_ by steady state analysis, the responses of the amount of complex at or close to equilibrium were plotted against the respective analyte concentrations and fitted to a 1:1 interaction model.

Supplemental Fig. 4 Surface plasmon resonance binding assessment to IL-2Rβγ. Surface plasmon resonance sensorgrams of (A, C, E) IgG-IL-2 and (B, D, F) IgG-(IL-2)_2_ binding to immobilized preformed (A, B) human, (C, D) cynomolgus, and (E, F) murine IL-2Rβγ-Fc. The biotinylated heterodimeric receptor was captured on a Biacore SA sensorchip via strepatvidin. To determine the *K*_D_ as well as association and dissociation rate constants by kinetic analysis, the recorded responses over time were fitted to a 1:1 interaction model. The dissociation rates were recorded for up to 600 s in order to observe a measurable decay of these high-affinity complexes. Dotted lines represent the recorded responses, straight lines a global fit of the data to a 1:1 interaction model.

Supplemental Fig. 5 Frequency of total, memory and naïve Tregs in normal cynomolgus blood. (A) Total Tregs are shown as the number per mm^3^ of blood (mean ± SD, *n* = 51). (B) Total Tregs and the division between memory and naïve Tregs are shown as the % of total CD4^+^ T cells (mean ± SD, *n* = 51). (C) The lack of correlation between Tregs/mm^3^ and Tregs as % CD4^+^ T cells in individual cynomolgus (*n* = 51). (D) Memory and naïve Tregs from individuals paired as % of CD4^+^ T cells (*n* = 51, *p*-value determined by a two-tailed paired *t*-test).

Supplemental Fig. 6 Transient IL-2-induced drop in circulating lymphocytes and NK cells. An IL-2-induced lymphopenia and simultaneous drop in NK cells occurred after dosing with Proleukin (400 pmol/kg, *n* = 3) and IgG-(IL-2)_2_ (34 pmol/kg, *n* = 5); vehicle (*n* = 6). (A) Total lymphocytes dropped 30–40% and returned to normal by 4–7 days post-dosing. (B) NK cells decreased 45–50% and returned to normal 4–7 days after dosing.

Supplemental Fig. 7 Effect of *in vivo* treatment on naïve Treg number and frequency. (A) Dose-dependent increases in naive Treg are compared as fold changes in Tregs/mm^3^ blood and (B) as increases in naïve Tregs as the % of CD4^+^ cells; baseline naïve Tregs averaged 1.5% as indicated. Group sizes were: IgG-IL-2 (all doses, *n* = 6); IgG-(IL-2)_2_ (11 and 34 pmol/kg, *n* = 5; 4 pmol/kg, *n* = 3); and Proleukin (40 and 400 pmol/kg, *n* = 3; 120 pmol/kg, *n* = 4).

Supplemental Fig. 8 Gating strategy for cell sorting and epigenetic analysis. (A) The separated subsets of CD4^+^ T cells, with naïve and memory Teff, total Tregs, CD25^hi^ and CD25^int^ Tregs. (B) CD25 expression in total Tregs before dosing. (C, D) Post-dosing the CD25 expression in Tregs is higher thus (E, F, G) allowing sorting of CD25^int^ Tregs from activated CD25^hi^ Tregs. (H, I, J) Memory and naïve Teff were sorted using the gating shown.
