# Supplementary material for: Sustained in vivo signaling by long-lived IL-2 induces prolonged increases of regulatory T cells
Source: J Autoimmun. 2015 Jan;56:66–80. doi: 10.1016/j.jaut.2014.10.002 (PMC4298360; doi:10.1016/j.jaut.2014.10.002)
Supplement: Supplemental Fig. 1 — IL-2 fusion protein schematics, Proleukin whole blood activity and in vivo dose conversions. (A, B) The human IgG-IL-2 and IgG-(IL-2)2 fusion proteins are illustrated: the V-region germline sequences have no known binding to human cells or tissues; the point mutations in the CH2 region of the human IgG1 molecule render the molecule effector-silent; knobs-into-holes technology was used to create monovalent IgG-IL-2. (C) The ability of Proleukin to stimulate pSTAT5a in human whole blood (n = 3 donors) is shown. (D) Maximal changes in CD4+ memory Teff cells are shown for Proleukin (400 pmol/kg, n = 3), IgG-IL-2 (157 pmol/kg, n = 6) and IgG-(IL-2)2 (34 pmol/kg, n = 5). (E) Using body surface area to translate dosage across species, the table shows the doses of Proleukin given to cynomolgus and their equivalent doses in humans (in IU/m2 or * per person). [file mmc1.pdf]

SUPPLEMENTARY FIGURE 1

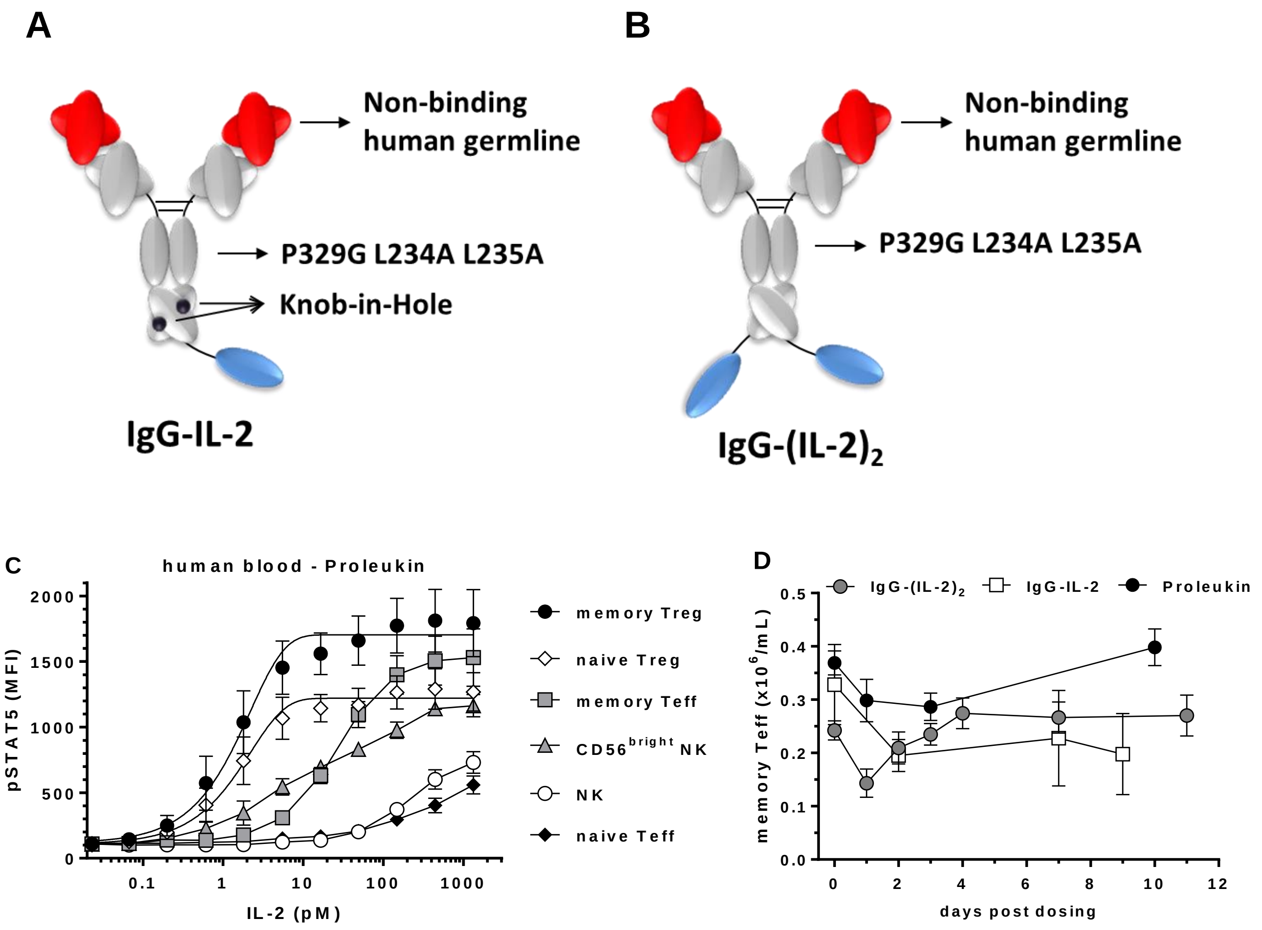

E

	Cynomolgus dose pmoles/kg (IU/kg)	Human equivalent dose (IU/m^2)
Proleukin	40 (10,000)	120,000
	120 (30,000)	360,000
	400 (100,000)	1,200,000
	800 (200,000)	4,500,000*
