# Supplementary figures and images for: Sustained in vivo signaling by long-lived IL-2 induces prolonged increases of regulatory T cells
Source: J Autoimmun. 2015 Jan;56:66–80. doi: 10.1016/j.jaut.2014.10.002 (PMC4298360; doi:10.1016/j.jaut.2014.10.002)

# SUPPLEMENTARY FIGURE 2

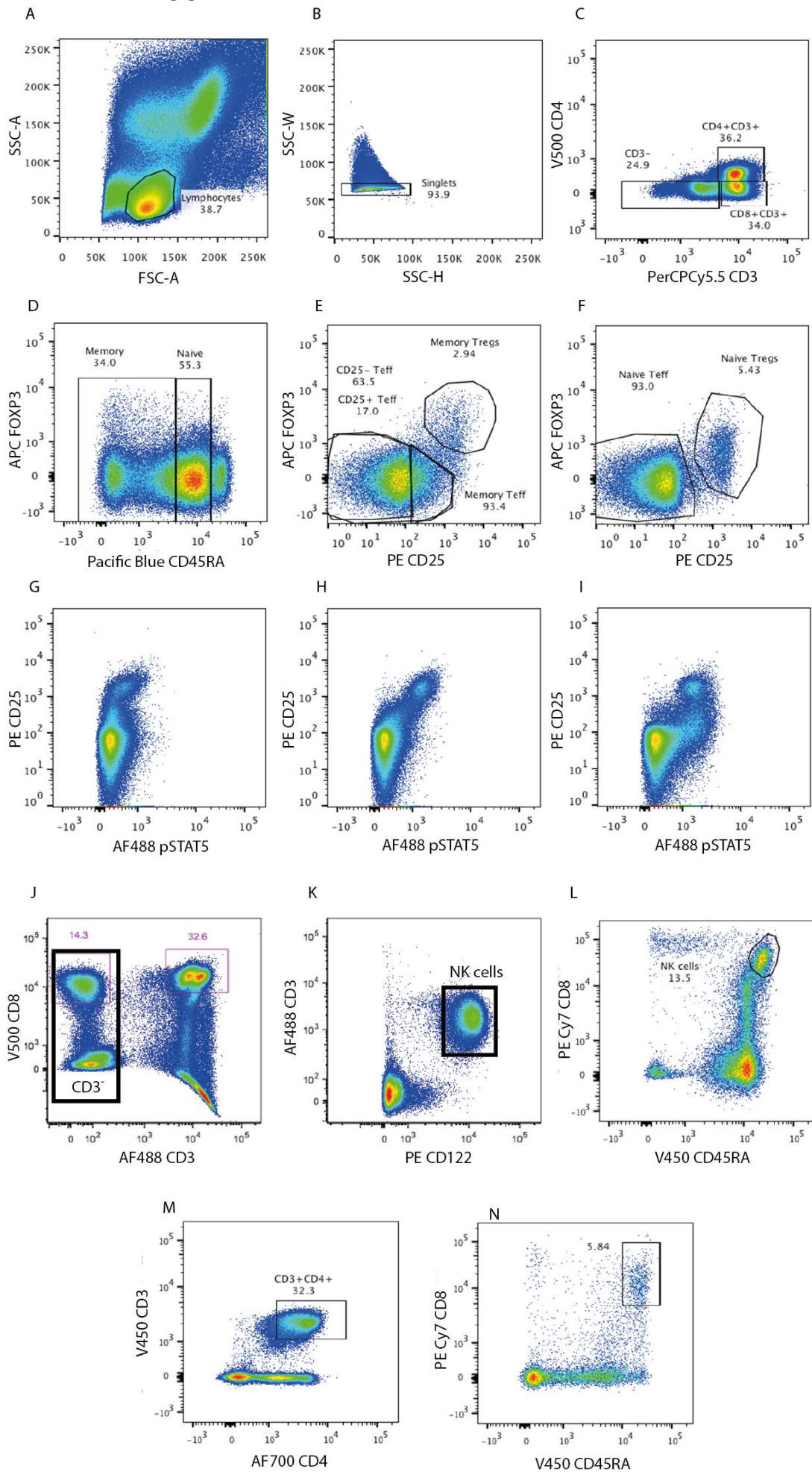

Supplement: Supplemental Fig. 2 — Flow cytometry gating of cynomolgus blood cell subsets. (A) Lymphocytes were isolated and (B) refined into single cells, a step performed during analysis of all tubes. (C) For intracellular staining, CD3+ T cells were split into CD4+ and CD4−CD8+ subsets (in the ex vivo tube CD4+ T cells were gated without CD3). (D) CD3+CD4+ T cells were separated into memory (CD45RA− and CD45RAintermediate) and naïve (CD45RA+) subsets. CD45RAintermediate cells were included in the memory gate because analyses of this “CD45RA transitional subset” revealed that molecules such as CD25, FOXP3 and Ki-67 had expression patterns more similar to CD45RA− as compared to CD45RA+ CD4+ T cells. Cells expressing very high levels of CD45RA were excluded from analysis: they were not present in all animals and showed no response to IL-2. Further investigation of the cells expressing high levels of CD45RA was performed: (M) CD3+CD4+ T cells were gated and (N) we found that CD8 was co-expressed on these cells. (E, F) CD3+CD4+ T cells not expressing high levels of levels of CD45RA were separated into Tregs and Teff in memory and naïve subsets for analyses. (E) CD25+ memory Teff are also shown. (G, H, I) The pSTAT5a status of total CD4+ memory T cells are shown responding in vitro to no, 5.7 pM and 570 pM IgG-(IL-2)2 demonstrating that the subset of memory cells expressing the highest levels of CD25 require lower concentrations of IL-2 to stimulate pSTAT5a increases. (J) Using the surface panel, CD3+CD8+ T cells were gated as were CD3−CD8+ NK cells. (K) The CD3− population is shown, identifying the CD3−CD8+ NK cells as CD122+. (L) The CD3− population is shown in demonstrating that CD8+CD3− cells are highest for CD45RA expression, allowing our identification of CD3−CD45RAhi cells as NK cells in our pSTAT5a intracellular panel. CD16 was used as a marker in the surface panel but showed heterogenous staining in the CD3−CD8+CD122+ subset across animals and was hence not used as an NK marker (data not shown) [file mmc2.pdf]

SUPPLEMENTARY FIGURE 4

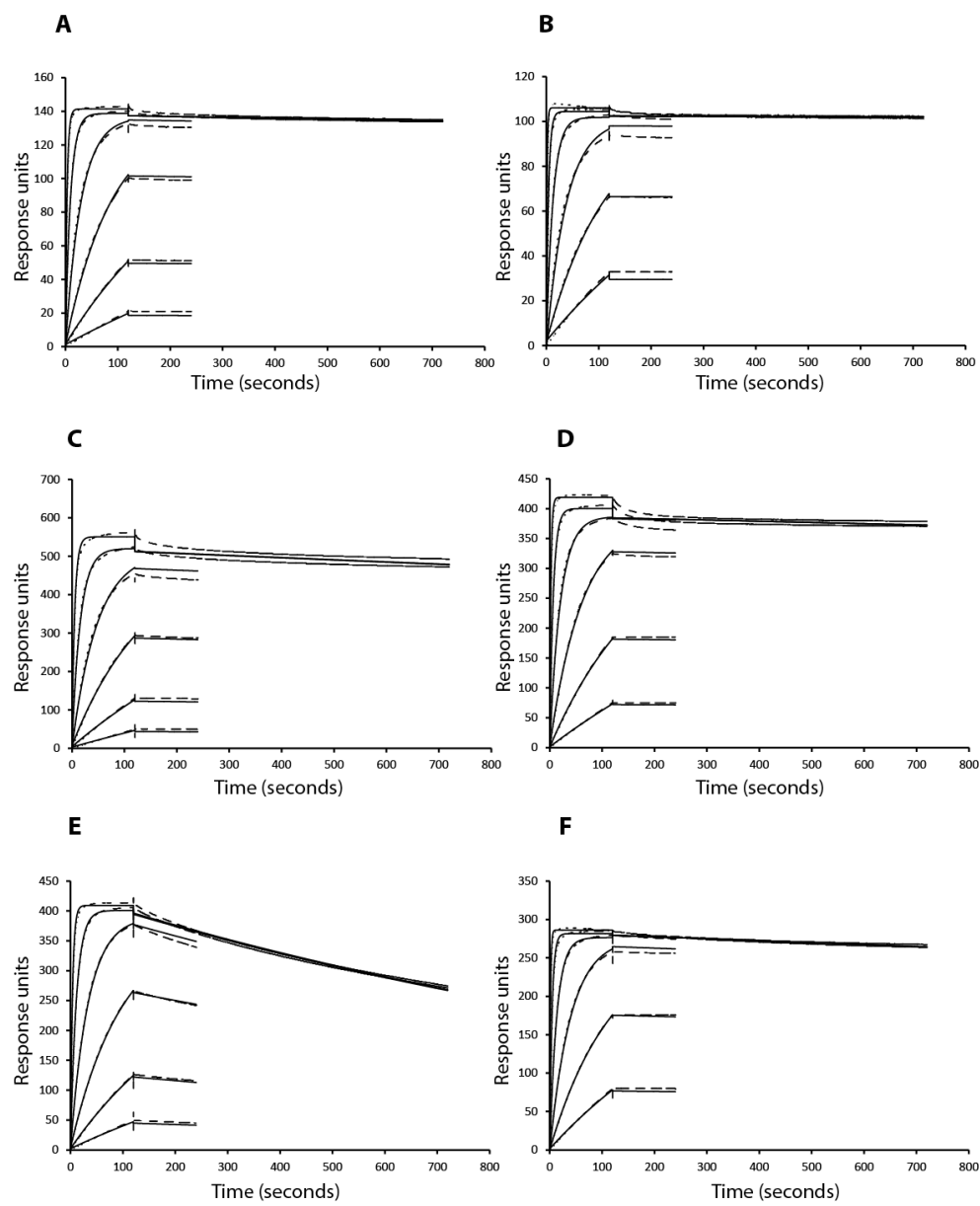

Supplement: Supplemental Fig. 4 — Surface plasmon resonance binding assessment to IL-2Rβγ. Surface plasmon resonance sensorgrams of (A, C, E) IgG-IL-2 and (B, D, F) IgG-(IL-2)2 binding to immobilized preformed (A, B) human, (C, D) cynomolgus, and (E, F) murine IL-2Rβγ-Fc. The biotinylated heterodimeric receptor was captured on a Biacore SA sensorchip via strepatvidin. To determine the KD as well as association and dissociation rate constants by kinetic analysis, the recorded responses over time were fitted to a 1:1 interaction model. The dissociation rates were recorded for up to 600 s in order to observe a measurable decay of these high-affinity complexes. Dotted lines represent the recorded responses, straight lines a global fit of the data to a 1:1 interaction model. [file mmc4.pdf]

SUPPLEMENTARY FIGURE 5

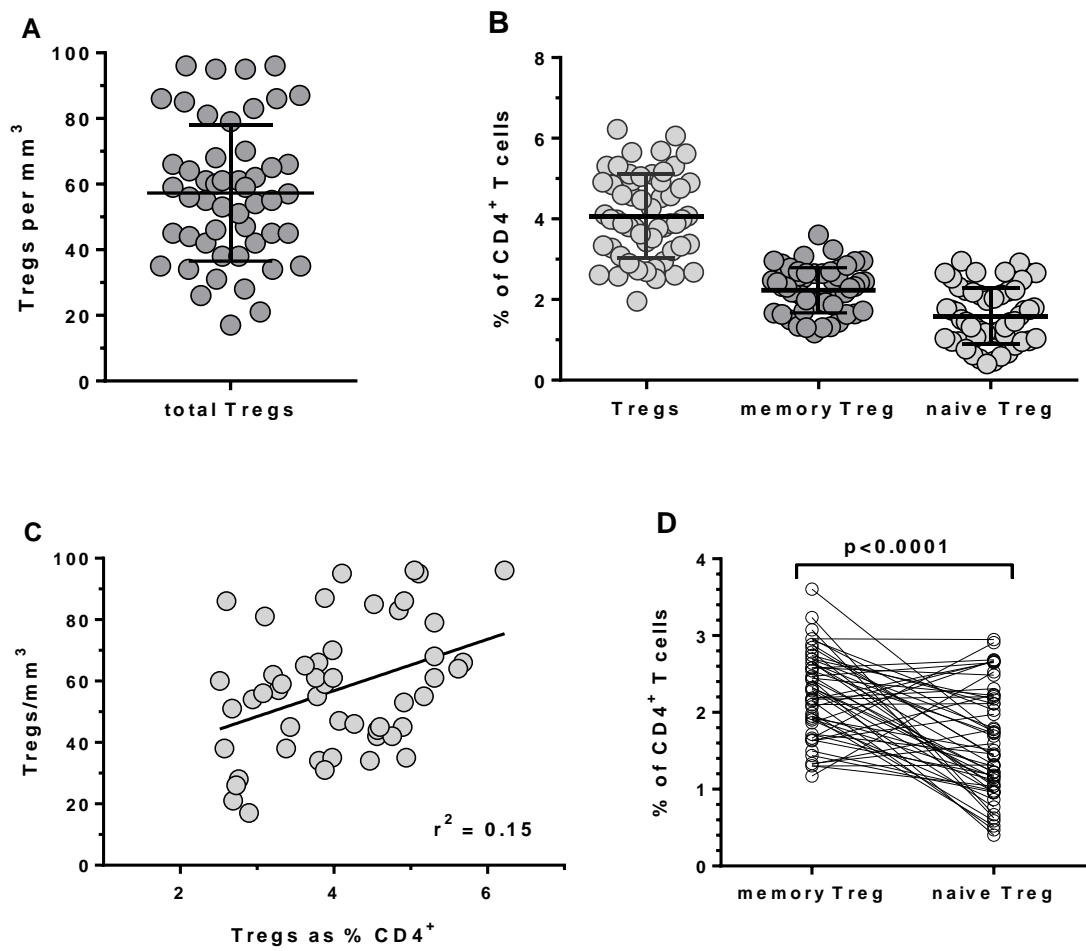

Supplement: Supplemental Fig. 5 — Frequency of total, memory and naïve Tregs in normal cynomolgus blood. (A) Total Tregs are shown as the number per mm3 of blood (mean ± SD, n = 51). (B) Total Tregs and the division between memory and naïve Tregs are shown as the % of total CD4+ T cells (mean ± SD, n = 51). (C) The lack of correlation between Tregs/mm3 and Tregs as % CD4+ T cells in individual cynomolgus (n = 51). (D) Memory and naïve Tregs from individuals paired as % of CD4+ T cells (n = 51, p-value determined by a two-tailed paired t-test). [file mmc5.pdf]

SUPPLEMENTARY FIGURE 6

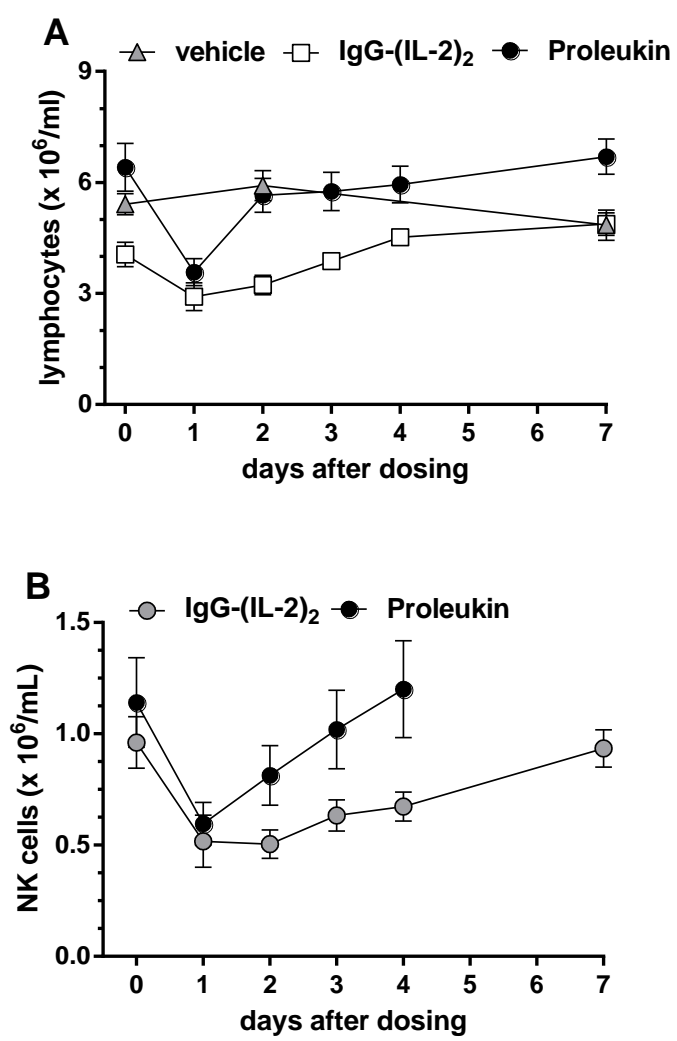

Supplement: Supplemental Fig. 6 — Transient IL-2-induced drop in circulating lymphocytes and NK cells. An IL-2-induced lymphopenia and simultaneous drop in NK cells occurred after dosing with Proleukin (400 pmol/kg, n = 3) and IgG-(IL-2)2 (34 pmol/kg, n = 5); vehicle (n = 6). (A) Total lymphocytes dropped 30–40% and returned to normal by 4–7 days post-dosing. (B) NK cells decreased 45–50% and returned to normal 4–7 days after dosing. [file mmc6.pdf]

## SUPPLEMENTARY FIGURE 7

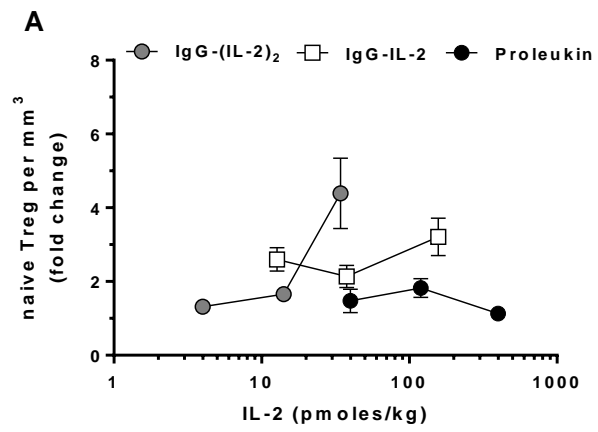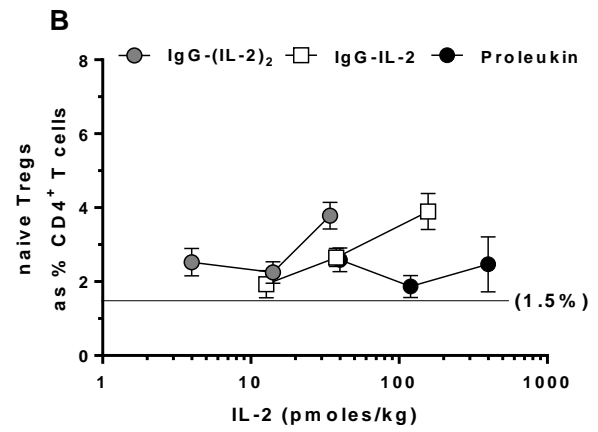

Supplement: Supplemental Fig. 7 — Effect of in vivo treatment on naïve Treg number and frequency. (A) Dose-dependent increases in naive Treg are compared as fold changes in Tregs/mm3 blood and (B) as increases in naïve Tregs as the % of CD4+ cells; baseline naïve Tregs averaged 1.5% as indicated. Group sizes were: IgG-IL-2 (all doses, n = 6); IgG-(IL-2)2 (11 and 34 pmol/kg, n = 5; 4 pmol/kg, n = 3); and Proleukin (40 and 400 pmol/kg, n = 3; 120 pmol/kg, n = 4). [file mmc7.pdf]

SUPPLEMENTARY FIGURE 8

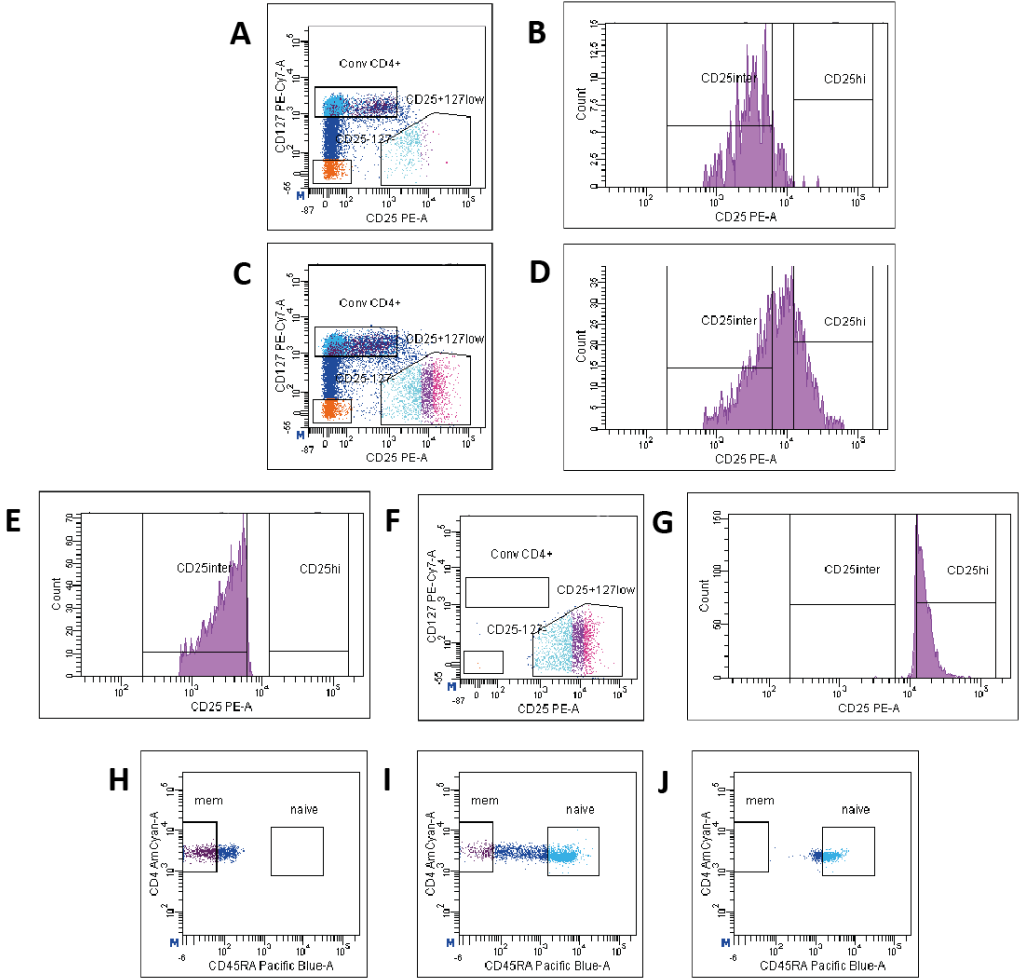

Supplement: Supplemental Fig. 8 — Gating strategy for cell sorting and epigenetic analysis. (A) The separated subsets of CD4+ T cells, with naïve and memory Teff, total Tregs, CD25hi and CD25int Tregs. (B) CD25 expression in total Tregs before dosing. (C, D) Post-dosing the CD25 expression in Tregs is higher thus (E, F, G) allowing sorting of CD25int Tregs from activated CD25hi Tregs. (H, I, J) Memory and naïve Teff were sorted using the gating shown. [file mmc8.pdf]
