# Supplementary material for: Sustained in vivo signaling by long-lived IL-2 induces prolonged increases of regulatory T cells
Source: J Autoimmun. 2015 Jan;56:66–80. doi: 10.1016/j.jaut.2014.10.002 (PMC4298360; doi:10.1016/j.jaut.2014.10.002)
Supplement: Supplemental Fig. 3 — Surface plasmon resonance binding assessment to IL-2Rα. Surface plasmon resonance sensorgrams of (A, C, E) IgG-IL-2 and (B, D, F) IgG-(IL-2)2 binding to immobilized (A, B) human, (C, D) cynomolgus, and (E, F) murine IL-2Rα. The receptor was chemically immobilized by amine coupling on a Biacore CM5 sensorchip. To determine the KD by steady state analysis, the responses of the amount of complex at or close to equilibrium were plotted against the respective analyte concentrations and fitted to a 1:1 interaction model. [file mmc3.pdf]

SUPPLEMENTARY FIGURE 3

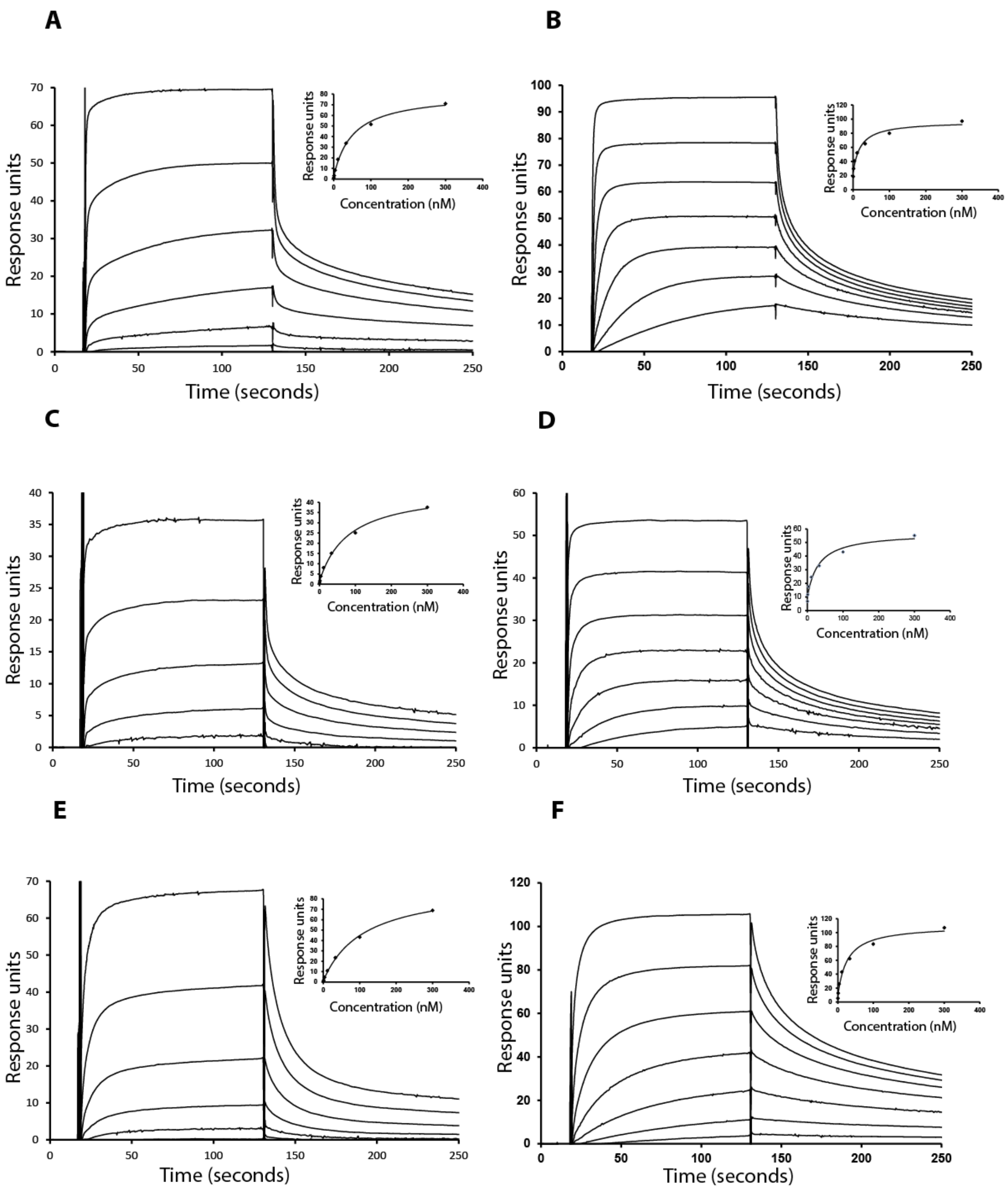

Construct	IL-2Rα	K_D steady state (nM)
IgG-IL-2	human	15
	cynomolgus	30
	murine	110
IgG-(IL-2)$_2$	human	5
	cynomolgus	15
	murine	30
